# Impaired cortical actin dynamics *via* lanosterol-dependent HMG-CoA reductase downregulation mediates IFN-α-induced mast cell stabilization

**DOI:** 10.1016/j.jbc.2026.113161

**Published:** 2026-05-16

**Authors:** Rema Naskar, Liu Ye, Sanu Karan, Kwan Soo Hong, Youngil Yeom, Inkyu Hwang

**Affiliations:** 1 Laboratory of Immunology and Immunopharmacology, College of Pharmacy, Chungnam National University, Daejeon, Republic of Korea; 2 Center for Target-to-Therapeutics Research, Korea Basic Science Institute, Cheongju, Republic of Korea; 3 Personalized Genomic Medicine Research Center, Korea Research Institute of Bioscience and Biotechnology (KRIBB), Daejeon, Republic of Korea

**Keywords:** interferon, mast cell, endoplasmic-reticulum-associated protein degradation (ERAD), isoprenoid, actin, HMG-CoA reductase, lanosterol, CYP51A1, mevalonate pathway: anaphylaxis

## Abstract

Independent studies have reported that interferon-β induces CYP51A1 downregulation leading to lanosterol (LAN) accumulation; that LAN triggers HMG-CoA reductase (HMGCR) downregulation; and that statin-mediated HMGCR inhibition suppresses mast cell activation. Building on these findings, we investigated whether the known mast cell–stabilizing effect of IFN-α/β is driven by this LAN-induced HMGCR downregulation. In bone marrow-derived mast cells (BMMCs), interferon-α (IFN-α) indeed induced HMGCR downregulation *via* both ubiquitin-mediated protein degradation and transcriptional suppression, alongside CYP51A1 downregulation and LAN accumulation. Cotreatment with terbinafine, a squalene epoxidase inhibitor used to prevent LAN synthesis, restored HMGCR expression to control levels and resulted in full recovery of bone marrow-derived mast cell function. Cotreatment with specific intermediates of the mevalonate (MVA) pathway (*i.e.*, mevalonolactone, farnesol, and geranylgeraniol) also counteracted the mast cell–stabilizing effect of IFN-α, without altering the HMGCR and CYP51A1 expression levels. These results indicate that attenuating the MVA pathway *via* LAN-induced HMGCR downregulation is directly responsible for the observed IFN-α-induced mast cell stabilization. Notably, IFN-α compromised the cortical actin dynamics necessary for high-affinity IgE receptor-induced LAT-PLC-γ1 signalosome assembly. These dynamics and signalosome assembly were also fully restored by cotreatment with terbinafine or the MVA pathway intermediates, as well as by the F-actin-destabilizing agent cytochalasin D. These findings were validated *in vivo* using a mouse model of passive cutaneous anaphylaxis and *ex vivo* mast cell degranulation assays. Together, our study reveals a previously unrecognized mechanism of immune regulation in which IFN-α stabilizes mast cells by impairing cortical actin dynamics through the CYP51A1-LAN-HMGCR axis.

Mast cells are long-lived tissue-resident cells that play a crucial role in defense against parasitic infection and the pathogenesis of allergy and anaphylaxis. The high-affinity IgE receptor (FcεRI) is integral to antigen (Ag)-induced mast cell activation, leading to the release of secretory granules (SGs) containing various inflammatory mediators and the *de novo* synthesis of cytokines (1).

Type I interferons (IFN-I) are a family of structurally related cytokines that signal through a common receptor designated as IFN-α/β receptor (IFNAR). Although originally recognized as antiviral and proinflammatory cytokines, they exert pleiotropic effects on the immune system that vary depending on the cell type and the stage of the immune response (2). The regulatory role of IFN-I in mast cell activation has been also documented (3, 4, 5). This regulatory effect is further supported by their therapeutic benefits in treating mastocytosis—a disease characterized by uncontrolled mast cell activation and proliferation—through a cytostatic mechanism of action (6).

HMG-CoA reductase (HMGCR) is the enzyme that catalyzes the conversion of HMG-CoA to mevalonate (MVA), the rate-limiting step of the MVA pathway—a metabolic pathway best known for synthesizing the precursors for sterol biosynthesis (7). The expression of HMGCR is tightly controlled (8). Under sterol-rich conditions, the cleavage of the N-terminal cytosolic domain of sterol regulatory element-binding protein-2 (SREBP-2) at the Golgi apparatus, which is required for SREBP-2 to enter the nucleus and activate HMGCR gene transcription, is inhibited. HMGCR expression is also regulated at the protein level. Specific sterols, particularly C4-dimethylated sterols represented by lanosterol (LAN) and 24,25-dihydrolanosterol (DHLAN), mediate the interaction between HMGCR and the INSIG/gp78/VCP complex at the endoplasmic reticulum (ER). This interaction promotes HMGCR ubiquitination and its subsequent degradation *via* the ER-associated degradation (ERAD) pathway.

The MVA pathway serves as the metabolic route for the synthesis of isopentenyl pyrophosphate and dimethylallyl pyrophosphate from acetyl-CoA. These five-carbon building blocks are essential for the synthesis of a vast array of sterol—including ergosterol, phytosterol, cholesterol, and common precursors such as LAN—and nonsterol isoprenoids, such as farnesyl pyrophosphate (FPP), geranylgeranyl pyrophosphate (GGPP), and squalene (SQL) (Fig. 1*A*) (7). The importance of the MVA pathway in regulating immune responses, including IgE-mediated mast cell activation, has been revealed in studies using statins, which are competitive inhibitors of HMGCR and are widely used as hypercholesterolemia medications (9, 10, 11). These studies highlight that the deficiencies in FPP and GGPP, and the consequent impairment of small GTPase prenylation (including the Rab, Rho, and Ras families), are central to statin-mediated immune regulation.

**Figure 1:**
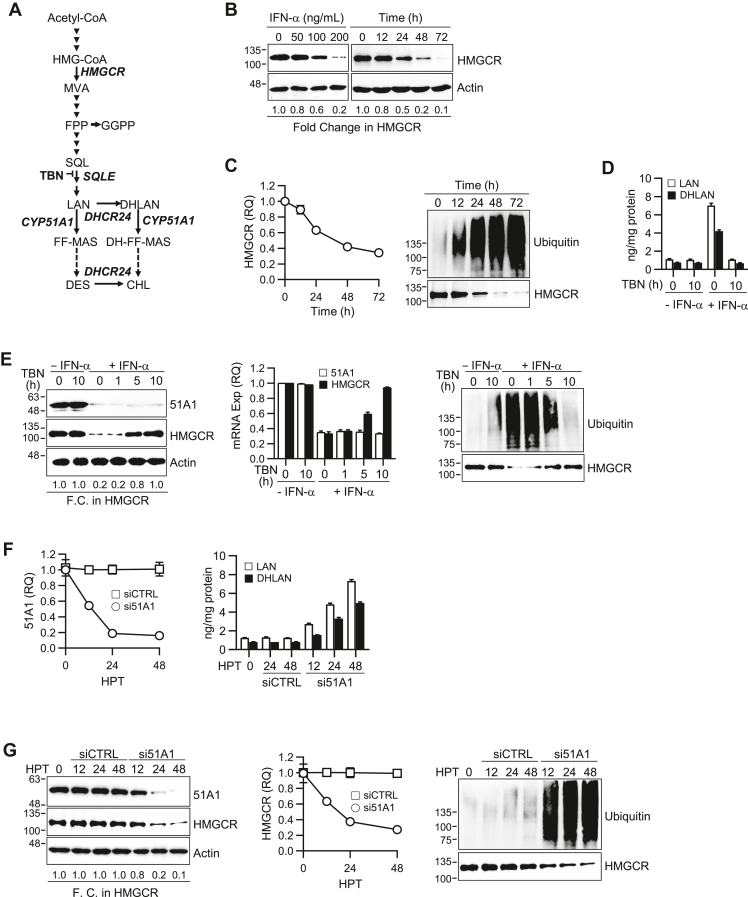
**IFN-α downregulates HMG-CoA reductase by inducing lanosterol accumulation *via* CYP51A1 repression.***A*, the mevalonate (MVA) and cholesterol biosynthesis pathways. *B*, IFN-α-induced HMGCR downregulation. Cell lysates prepared from bone marrow-derive mast cells (BMMCs), treated with vehicle (1 × PBS) or IFN-α at varying concentrations for 48 h (*left*) or at 200 ng/ml for the indicated durations (*right*), were subjected to Western blot (WB) analysis. HMGCR band intensities, normalized to β-actin, are shown as fold change relative to the control. *C*, IFN-α-induced reduction of HMGCR mRNA expression and enhancement of protein ubiquitination. BMMCs treated with or without IFN-α (200 ng/ml) for the indicated durations were analyzed for their HMGCR mRNA expression (*left*) and protein ubiquitination (*right*). Protein extracts were subjected to immunoprecipitation with an anti-HMGCR Ab prior to immunoblotting with anti-ubiquitin or anti-HMGCR Ab. *D*, counteracting effect of terbinafine on IFN-α-induced lanosterol/dihydrolanosterol accumulation. HPLC analyses were performed on BMMCs cultured in the presence or absence of IFN-α (200 ng/ml) for 48 h, with vehicle (DMSO) alone or TBN (20 μM) added for the indicated durations during the incubation. The graph shows cellular levels of LAN and DHLAN normalized to total protein in the cell lysates (mg). *E*, counteracting effect of TBN on IFN-α-induced HMGCR downregulation. BMMCs treated with or without IFN-α and TBN as in (*D*) were analyzed by immunoblotting for the levels of CYP51A1 (denoted as 51A1) and HMGCR expression (*left*); by RT-qPCR for the levels of HMGCR and CYP51A1 mRNA expression (*center*); for the level of HMGCR ubiquitination (*right*). *F*, siCYP51A1-mediated CYP51A1 silencing and LAN/DHLAN accumulation. BMMCs cultured for the indicated h posttransfection (HPT) with control siRNA (siCTRL) or siCYP51A1 (denoted as si51A1) were analyzed by RT-qPCR for CYP51A1 mRNA expression (*left*) and by HPLC for cellular LAN/DHLAN levels (*right*). *G*, siCYP51A1-induced downregulation of HMGCR. BMMCs transfected with the siRNA as in (*F*) were analyzed by immunoblotting for the levels of CYP51A1 and HMGCR (*left*); by RT-qPCR for the level of HMGCR mRNA expression (*center*); for the level of HMGCR ubiquitination (*right*). Data are presented as the mean ± SD of one representative experiment from three independent trials. qPCR and HPLC analyses were conducted in triplicate and duplicate, respectively. WB images are representative of three independent experiments. BMMC, bone-marrow-derived mast cell; DHLAN, 24,25-dihydrolanosterol; DMSO, dimethyl sulfoxide; HMGCR, HMG-CoA reductase; IFN-α, interferon-α; LAN, lanosterol; PBS, phosphate-buffered saline; RT-qPCR, reverse transcription quantitative PCR; siCYP51A1, CYP51A1-targeted siRNA; TBN, terbinafine.

Recently, a study by Araldi *et al.* using bone marrow-derived macrophages demonstrated that interferon-β induces LAN accumulation by suppressing the transcription of the CYP51A1 gene (12), which encodes an enzyme that converts LAN to FF-MAS or DHLAN to DH-FF-MAS, *via* a demethylation reaction at C14 during cholesterol synthesis (Fig. 1*A*) (7, 13). Based on this literature, we investigated whether the known mast cell-stabilizing effects of IFN-Ι are attributed to the HMGCR downregulation and consequent MVA pathway inhibition, driven by LAN accumulation following the transcriptional suppression of CYP51A1, in a manner similar to statin treatment.

## Results

### Interferon-α (IFN-α) downregulates HMGCR by inducing LAN accumulation *via* CYP51A1 repression

Consistent with the report by Araldi *et al.* (12), interferon-α (IFN-α) reduced CYP51A1 mRNA and protein expression in a concentration- and time-dependent manner, leading to the accumulation of LAN and DHLAN in bone marrow-derived mast cells (BMMCs) (Fig. S1). Notably, IFN-α also reduced HMGCR protein expression in a dose- and time-dependent manner (Fig. 1*B*). This was accompanied by a decrease in its mRNA levels and a concomitant increase in protein ubiquitination (Fig. 1*C*).

To determine the contribution of LAN/DHLAN accumulation to HMGCR downregulation, we cotreated IFN-α-pretreated BMMCs with terbinafine (TBN), an inhibitor of squalene epoxidase (SQLE) that prevents LAN synthesis (Fig. 1*A*) (14). TBN treatment completely abrogated the IFN-α-induced accumulation of LAN/DHLAN (Figs. 1*D* and S1). Notably, TBN cotreatment also fully restored HMGCR expression within 10 h, without altering CYP51A1 expression levels (Fig. 1*E*). This restoration was linked to a recovery of its mRNA levels and a reduction in protein ubiquitination (Fig. 1*E*).

To confirm the causal relationship between the transcriptional suppression of CYP51A1 and HMGCR downregulation, we transfected the cells with CYP51A1-targeted siRNA (siCYP51A1). Silencing of CYP51A1, which reduced its mRNA levels by over 80% within 24 h, also led to a roughly 4.5-fold increase in LAN and DHLAN concentrations (Figs. 1*F* and S1). Furthermore, CYP51A1 knockdown reduced HMGCR protein expression by approximately 80%, paralleling a decrease in its mRNA levels and an increase in protein ubiquitination (Fig. 1*G*).

### Both specific MVA pathway intermediates and the LAN-accumulation inhibitor TBN rescue mast cell functions impaired by IFN-α or CYP51A1 silencing

**Figure 2:**
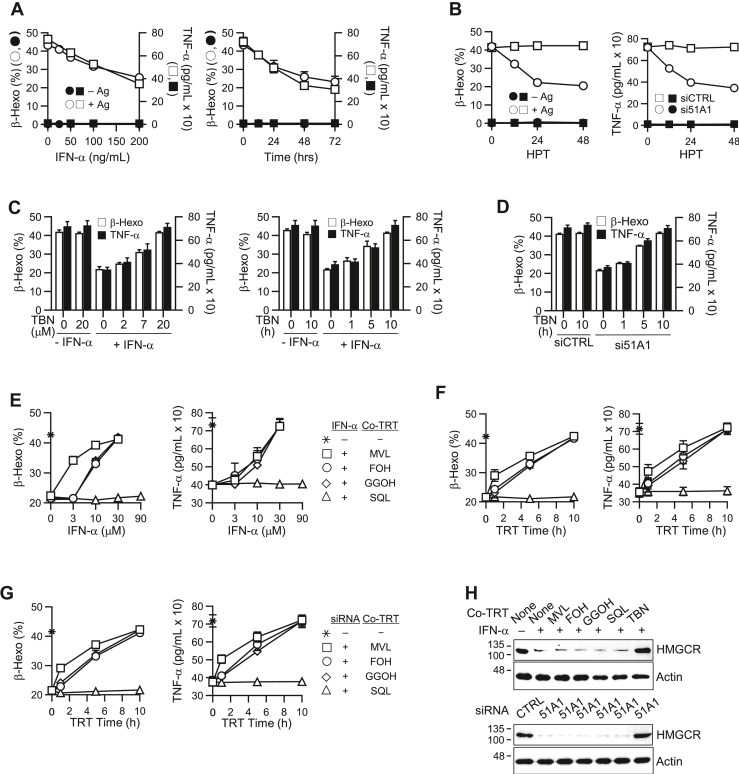
**Both specific mevalonate pathway intermediates and the lanosterol-accumulation inhibitor, terbinafine, rescue mast cell functions impaired by IFN-α or CYP51A1 silencing.***A*, suppression of FcεRI-mediated degranulation and TNF-α production by IFN-α. BMMCs, sensitized overnight with IgE during treatment with IFN-α at varying concentrations for 48 h (*left*) or at 200 ng/ml for the indicated durations (*right*), were challenged ± Ag. The levels of β-hexosaminidase release and TNF-α production are plotted. *B*, suppression of degranulation and TNF-α production by CYP51A1 silencing. BMMCs, sensitized overnight with IgE during the final stage of the indicated posttransfection periods (h), were challenged ± Ag. *C* and *D*, rescue of IFN-α- or siCYP51A1-suppressed BMMC function by TBN. BMMCs were treated with TBN during a 48-h incubation ± IFN-α (*C*), or during a 24-h incubation posttransfection with the siRNA (*D*). TBN was added at varying concentrations for the final 10 h (*C*, *left*) or at 20 μM for the indicated durations (*C*, *right*; D). BMMCs were sensitized and challenged as in (*A*). *E* and *F*, rescue of IFN-α-suppressed BMMC function by MVA pathway intermediates. BMMCs were treated with MVL, FOH, GGOH, or SQL at varying concentrations for the final 10 h (*E*), or at fixed concentrations (30 μM for MVL, FOH, and GGOH; 90 μM for SQL) for the indicated duration (*F*) during a 48-h incubation ± IFN-α. *G*, rescue of siCYP51A1-suppressed BMMC function by MVA pathway intermediates. BMMCs were treated with MVL, FOH, GGOH, or SQL at the concentration described in (*F*) for the indicated durations during the final stage of a 24-h culture period post-siRNA transfection. *H*, no rescue of IFN-α- or siCYP51A1-induced HMGCR downregulation by MVA pathway intermediates. Cell lysates prepared from BMMCs, treated as described in (*F*) or (*G*), were subjected to immunoblotting. Data are presented as the mean ± SD of one representative experiment from three independent trials. Each assay was conducted in duplicate. BMMC, bone-marrow-derived mast cell; FcεRI, high-affinity IgE receptor; FOH, farnesol; GGOH, geranylgeraniol; IFN-α, interferon-α; IgE, immunoglobulin E; MVA, mevalonate; MVL, mevalonolactone; TNF-α, tumor necrosis factor α; siCYP51A1, CYP51A1-targeted siRNA; siRNA, small interfering RNA; SQL, squalene; TBN, terbinafine.

IFN-α treatment and CYP51A1 silencing reduced the level of FcεRI-mediated degranulation (measured by β-hexosaminidase release) and tumor necrosis factor α (TNF-α) production in BMMCs in a dose- and time-dependent manner (Fig. 2, *A* and *B*). Notably, cotreatment with TBN restored these levels, in a dose- and time-dependent manner, achieving full recovery within 10 h at 20 μM (Fig. 2, *C* and *D*). This suggests that the impaired mast cell function was primarily attributable to the accumulation of LAN/DHLAN.

Cotreatment of IFN-α-pretreated BMMCs with mevalonolactone (MVL)—the lactone form of MVA (the product of HMGCR)—restored FcεRI-mediated β-hexosaminidase release and TNF-α production in a dose- and time-dependent manner. Near-complete recovery (>95%) was achieved within 10 h at a concentration of 30 μM (Fig. 2, *E* and *F*). In addition, cotreatment with specific nonsterol isoprenoids derived from MVA, such as farnesol (FOH) and geranylgeraniol (GGOH), yielded similar restorative effects. In contrast, SQL, the immediate precursor of LAN, failed to rescue mast cell function at concentrations up to 90 μM. These results indicate that the impaired mast cell function results from the inhibition of the MVA pathway consequent to HMGCR downregulation.

MVL, FOH, and GGOH—but not SQL—also restored β-hexosaminidase release and TNF-α production in BMMCs transfected with siCYP51A1 in a time-dependent manner (Fig. 2*G*). As expected, and in contrast to TBN, this functional recovery occurred without the restoration of HMGCR expression, which remained suppresses in IFN-α-treated and siCYP51A1-transfected cells following these cotreatments (Fig. 2*H*). These findings indicate that MVA pathway inhibition is primarily responsible for the impaired mast cell function, effectively ruling out any unidentified noncanonical effects of HMGCR downregulation.

### IFN-α and CYP51A1 silencing impair FcεRI-mediated Ca^2+^ signaling by disrupting LAT-PLC-γ1 signalosome assembly *via* the CYP51A1-LAN-HMG CoA reductase axis

**Figure 3:**
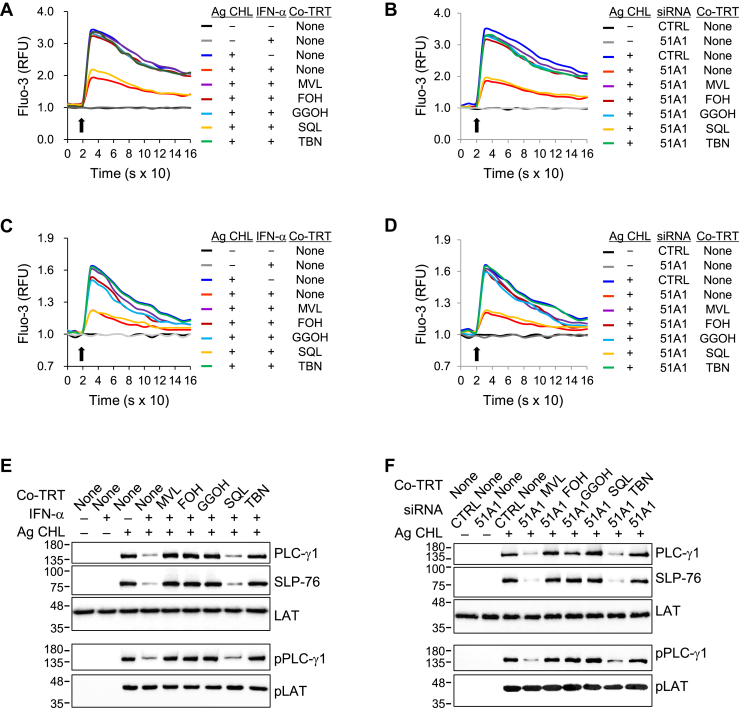
**IFN-α and CYP51A1 silencing impair FcεRI-mediated Ca^2+^ signaling by disrupting LAT-PLC-γ1 signalosome assembly *via* the CYP51A1-lanosterol-HMG CoA reductase axis.***A* and *B*, restoration of FcεRI-mediated Ca^2+^ influx impaired by IFN-α or siCYP51A1 *via* TBN and MVA pathway intermediates. BMMCs were sensitized and treated with MVL (30 μM), FOH (30 μM), GGOH (30 μM), SQL (90 μM), or TBN (20 μM) for the final 10 h of a 48-h IFN-α treatment (*A*) or a 24-h post-siRNA transfection period (*B*). Cells were then loaded with Fluo-3 and subjected to a flow cytometry-based calcium assay. The *arrow* indicates the time of Ag challenge. *C* and *D*, restoration of impaired intracellular Ca^2+^ mobilization by TBN and MVA pathway intermediates. BMMCs, treated as described above, were subjected to a calcium assay in a “Ca^2+^-free” buffer. Panels (*C*) and (*D*) show the effects of IFN-α treatment and siRNA transfection, respectively, both in the presence or absence of the indicated cotreatments. *E* and *F*, restoration of impaired LAT signalosome assembly by TBN and MVA pathway intermediates. BMMCs, treated as described in (*A* and *B*), were challenged ± Ag in a “Ca^2+^-free” buffer for 30 s prior to lysis. Cell lysates were subjected to immunoprecipitation with an anti-LAT mAb, followed by immunoblotting using the indicated mAbs. Panels (*E*) and (*F*) represent IFN-α treatment and siCYP51A1 transfection, respectively, each in the presence or absence of cotreatment. Ag, antigen; BMMC, bone-marrow-derived mast cell; FcεRI, high-affinity IgE receptor; FOH, farnesol; GGOH, geranylgeraniol; IFN-α, interferon-α; LAT, linker for activation of T cell; mAb, monoclonal antibody; MVA, mevalonate; MVL, mevalonolactone; PLC-γ1, phospholipase C-γ1; siRNA, small interfering RNA; SQL, squalene; TBN, terbinafine.

Both IFN-α treatment and CYP51A1 silencing markedly reduced FcεRI-mediated extracellular Ca^2+^ influx, a process essential for SG release (15). Consistent with our observation of mast cell degranulation and TNF-α production, cotreatment with TBN, MVL, FOH, and GGOH—but not SQL—effectively restored the extracellular Ca^2+^ entry suppressed by IFN-α and CYP51A1 silencing (Fig. 3, *A* and *B*). Furthermore, IFN-α and siCYP51A1 also reduced FcεRI-mediated intracellular Ca^2+^ mobilization from the ER, which precedes and triggers extracellular Ca^2+^ influx. This intracellular Ca^2+^ mobilization was also rescued by cotreatment with TBN, MVL or nonsterol isoprenoids (*i.e.*, FOH and GGOH) (Fig. 3, *C* and *D*).

The mobilization of intracellular Ca^2+^ depends on the activation of phospholipase C-γ1 (PLC-γ1), which occurs through the assembly of a multiprotein signaling complex (signalosome) centered on linker for activation of T cells (LAT) (15). We therefore examined how IFN-α and CYP51A1 siRNA affected FcεRI-mediated LAT signalosome assembly. Both IFN-α treatment and CYP51A1 siRNA markedly decreased the amounts of PLC-γ1 and the adapter protein SLP-76—which serves as a critical scaffold for assembling the downstream signaling proteins on the LAT platform (16)—that coprecipitated with LAT. The reduction in LAT-associated PLC-γ1 correlated with a significant decrease in PLC-γ1 phosphorylation at tyrosine 783 (Y783) (Fig. 3, *E* and *F*), a modification critical for PLC-γ1 activation and mediated by Syk upon its recruitment to the LAT complex (17). Consistent with our previous results, cotreatment with MVL, FOH, or GGOH almost completely restored the recruitment of PLC-γ1 and SLP-76 to LAT as well as the level of PLC-γ1 phosphorylated at Y783.

Notably, separate from their effects on the FcεRI-mediated LAT signalosome assembly, neither IFN-α nor CYP51A1 siRNA significantly altered the tyrosine phosphorylation of LAT (Fig. 3, *E* and *F*). This is particularly striking because LAT phosphorylation is a prerequisite for signalosome assembly, as it provides docking sites for SH2-domain-containing proteins, including PLC-γ1. The observation that the level of phosphorylated LAT remained unaffected prompted us to investigate upstream signaling events responsible for its phosphorylation, including the activation of Syk, Lyn, and the common Fcγ-chain (15). Consistent with our findings for LAT, neither IFN-α nor CYP51A1 siRNA altered the phosphorylation levels of Syk, Lyn, or Fcγ-chain upon FcεRI triggering—widely accepted indicators of their activation status (Fig. S2). Accordingly, cotreatment with MVL, FOH, or GGOH also had no noticeable effect on the phosphorylation status of these proteins.

### IFN-α and CYP51A1 silencing impair cortical actin dynamics *via* the CYP51A1-LAN-HMG CoA reductase axis

Several studies have demonstrated that mutation or silencing of genes involved in actin reorganization (*e.g.*, WASP and WAVE), or treatment with an F-actin stabilizing agent like jasplakinolide, impairs PLC-γ activation. This impairment arises specifically from defective LAT signalosome assembly, without affecting the activation of LAT and its upstream signaling proteins, such as Zap-70/Syk family protein tyrosine kinases (18, 19, 20, 21). These findings underline the critical role of dynamic cortical actin remodeling in TCR- and FcεRI-mediated PLC-γ1 activation. Given these reports and our own observations that IFN-α prevents FcεRI-mediated LAT signalosome assembly despite intact LAT phosphorylation, we investigated whether IFN-α alters the dynamics of the cortical actin cytoskeleton.

**Figure 4:**
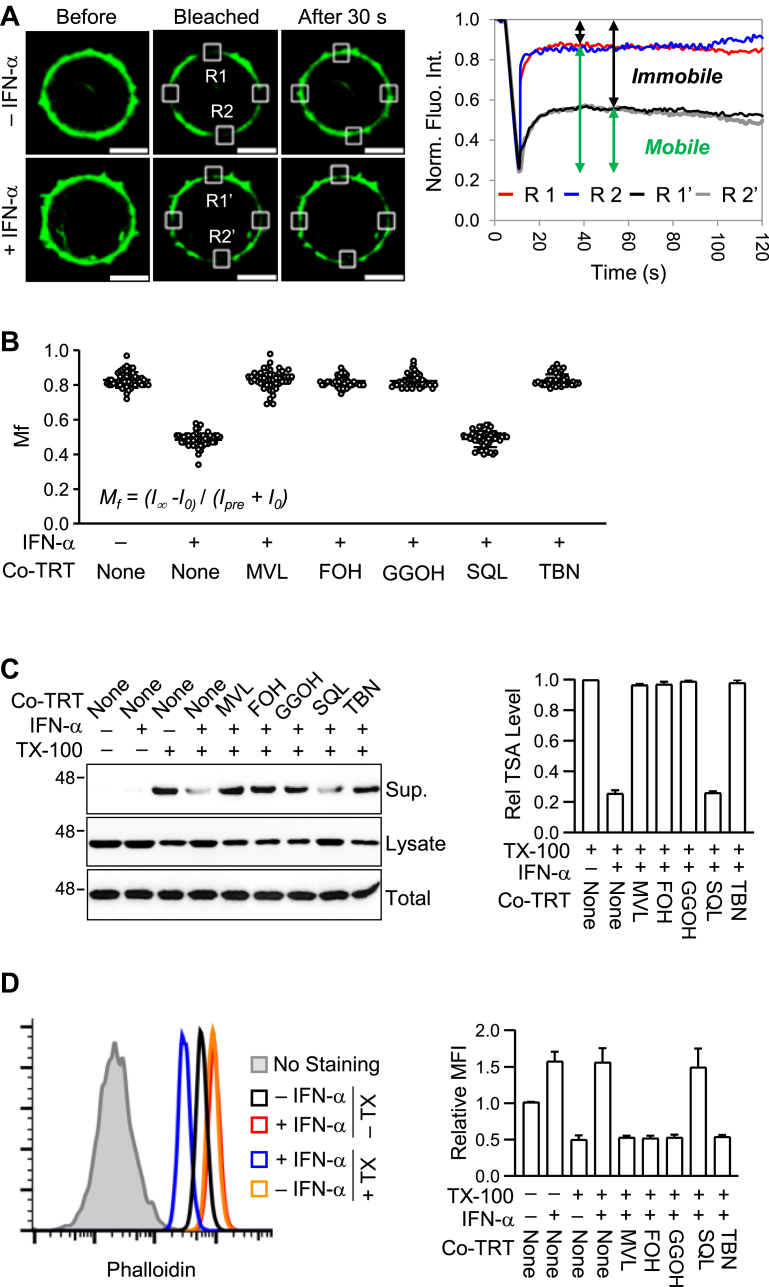
**IFN-α impairs cortical actin dynamics *via* the CYP51A1-lanosterol-HMG CoA reductase axis.***A* and *B*, restoration of Mf reduced by IFN-α *via* TBN and mevalonate pathway intermediates. Lifeact-expressing BMMCs were subjected to FRAP assays after a 48-h incubation ± 200 ng/ml IFN-α. BMMCs were captured before, immediately after, and 30 s after photobleaching (*A*, *left*). The scale bar represents 5 μm. Representative fluorescence recovery patterns over 110 s postphotobleaching are expressed as “normalized fluorescence intensities” in ROIs (*A*, *right*). Mobile fractions (M_f_) were determined from 48 ROIs (4 ROIs per cell) in BMMCs treated with vehicle (none), an MVA pathway product or TBN at the concentrations described in Figure 3*A* for the last 10 h of a 48-h incubation ± IFN-α. Individual data points are plotted alongside the mean ± SD (*B*). The Mf was calculated as described. *C*, restoration of Triton X100-soluble actin reduced by IFN-α *via* TBN and MVA pathway intermediates. BMMCs, treated as described above during a 48-h incubation ± IFN-α were washed with an actin-stabilizing buffer ± Triton X-100. Supernatants and cell lysates were subjected to immunoblotting with an anti-actin mAb as means to quantify Triton X-100-soluble actin (TSA) and Triton X-100-insoluble actin (TIA), respectively. Actin band intensities of the supernatants were normalized to the total (supernatant + cell lysate), and the relative changes compared to the control are shown. The data present the mean ± SD of three independent experiments. *D*, restoration of Triton X100-insoluble actin elevated by IFN-α *via* TBN and MVA pathway intermediates. BMMCs, treated and washed with the buffer ± Triton X-100 as described above, were fixed with PFA and stained with fluorescently labeled phalloidin for flow cytometry analyses. Representative histograms are shown. The mean fluorescence intensities (MFIs), relative to those of control BMMCs, are also presented as the mean ± SD from three experiments performed in duplicate. BMMC, bone-marrow-derived mast cell; IFN-α, interferon-α; MVA, mevalonate; TBN, terbinafine.

Fluorescence recovery after photobleaching assays using BMMCs expressing a Lifeact-GFP fusion protein revealed that fluorescence intensity in the photobleached region of interest (ROI) increased sharply at the onset of photorecovery, reaching a plateau within 20 s in control cells (Fig. 4*A*). However, both the initial recovery rate and the mobile fraction (Mf) were markedly reduced in IFN-α-treated BMMCs. Specifically, the average Mf across 48 ROIs in IFN-α-treated BMMCs—measured 30 s postbleaching—was approximately half that of untreated controls (Fig. 4*B*). These results indicate a significant impairment in the turnover and dynamics of cortical actin network following IFN-α treatment. Consistent with our previous findings, cotreatment with TBN or MVA pathway metabolites (MVL, FOH, and GGOH) restored the Mf in IFN-α-treated BMMCs to levels comparable to those of control cells.

The level of Triton X-100-soluble actin (TSA), detected in the supernatant after washing BMMCs with a Triton X-100-containing actin stabilization buffer, represents the cellular pool of globular actin plus small fragmented F-actin in cells (22). This TSA pool decreased by nearly 70% when BMMCs were treated with IFN-α (Fig. 4*C*) or transfected with siCYP51A1 (Fig. S3). Notably, this reduction was accompanied by a significant increase in the amount of insoluble F-actin remaining in the cell pellet. Cotreatment with MVL, FOH, GGOH, or TBN restored the TSA levels to those observed in control BMMCs.

Similar results were obtained when Triton X100-insoluble actin levels were assessed by flow cytometry using fluorescently labeled phalloidin. In control BMMCs, phalloidin fluorescence intensity decreased by approximately 50% after washing with the Triton X-100-containing buffer, reflecting the extraction of small fragmented F-actin pool. In contrast, the fluorescence intensity in IFN-α-treated (Fig. 4*D*) or siCYP51A1-transfected cells (Fig. S3) remained nearly unchanged. Notably, the baseline fluorescence intensities in these cells were significantly higher than that in control cells even before washing, suggesting an overall increase in total F-actin levels induced by IFN-α or CYP51A1 silencing. Taken together, these results indicate that IFN-α treatment and CYP51A1 silencing lead to significant F-actin stabilization and a reduction in the rate of F-actin turnover and remodeling.

### Cytochalasin D counteracts the mast cell-stabilizing effect of IFN-α and CYP51A1 silencing by restoring cortical actin dynamics

Cytochalasin D (CytD), at low nanomolar concentrations, binds specifically to the barbed end of F-actin, inhibiting F-actin elongation typically driven by barbed-end binding proteins such as Ena/VASP and formins (23). In addition, CytD remodels the cortical actin cytoskeleton, resulting in increased cell motility and reduced surface stiffness (24, 25). CytD has also been shown to rescue failed cytokinesis caused by the excessive accumulation of formin-based linear actin filaments in capping protein-deficient cells (26). Given CytD’s ability to modulate cellular F-actin dynamics, we investigated whether it could counteract the mast cell-stabilizing effects of IFN-α and CYP51A1 silencing.

**Figure 5:**
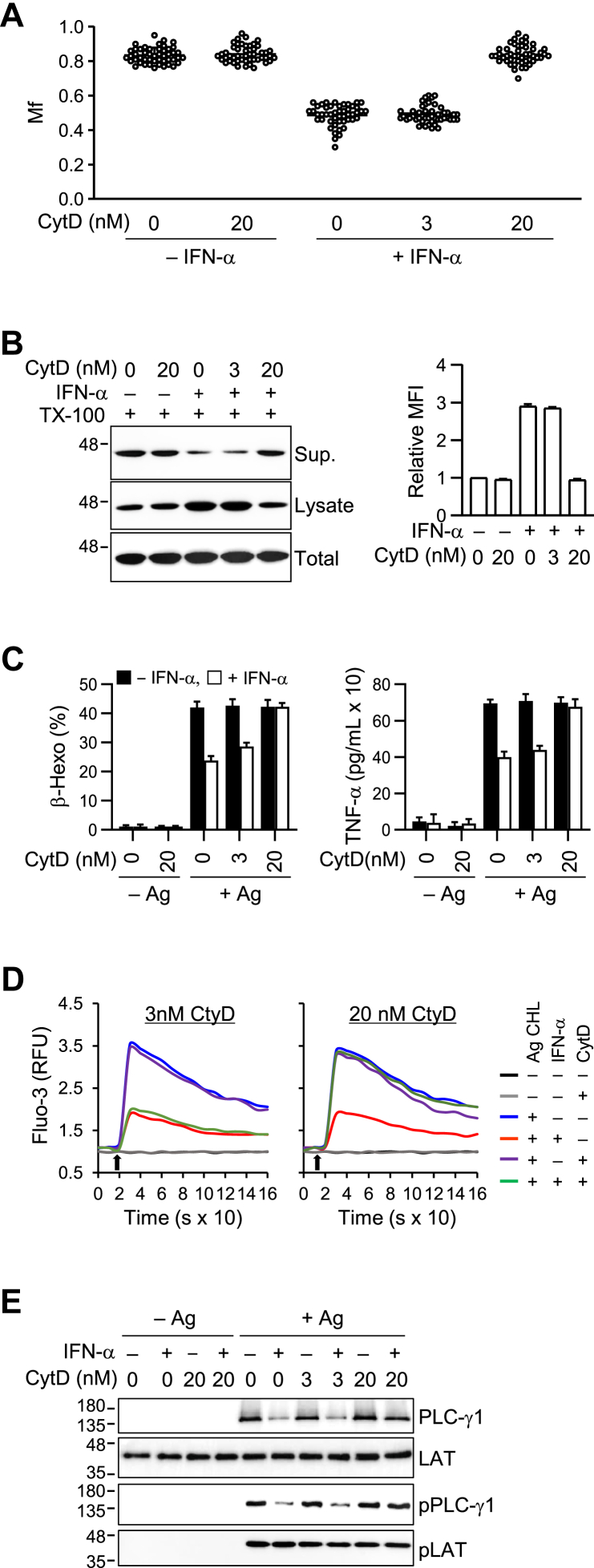
**Cytochalasin D counteracts the mast cell-stabilizing effect of IFN-α by restoring cortical actin dynamics.***A*, restoration of IFN-α-reduced mobile fraction (Mf) by cytochalasin D. BMMCs, incubated for 48 h in the presence or absence of 200 ng/ml IFN-α, were treated with CytD at the indicated concentration for 20 min immediately prior to the fluorescence recovery after photobleaching (FRAP) assay. *B*, normalization of IFN-α-induced changes in Triton X-100-soluble and Triton X100-insoluble actin levels by CytD. The TSA and TIS levels of BMMCs treated as described above were assessed using immunoblotting (*left*) and flow cytometry (*right*), as described in Figure 4, *C* and *E*, respectively. *C*, restoration of IFN-α-suppressed degranulation and TNF-α production by CytD. BMMCs, sensitized overnight with IgE during a 48-h incubation ± IFN-α, were treated with CytD immediately before challenge ± Ag. *D*, restoration of IFN-α-impaired Ca^2+^ influx by CytD. BMMCs, treated and sensitized as described above, were subjected to the Ca^2+^ assay in a ‘Ca^2+^-containing” buffer. *E*, restoration of IFN-α-impaired LAT-PLC-γ1 complex formation by CytD. BMMCs, treated and sensitized as described above, were challenged ± Ag in a “Ca^2+^-free” buffer. Cell lysates were subjected to immunoprecipitation with an anti-LAT mAb, followed by immunoblotting using the indicated Abs. BMMC, bone-marrow-derived mast cell; CytD, cytochalasin D; IFN-α, interferon-α; IgE, immunoglobulin E; LAT, linker for activation of T cell; mAb, monoclonal antibody; PLC-γ1, phospholipase C-γ1; TIS, Triton X100-insoluble actin; TSA, Triton X-100-soluble actin.

At a concentration of 20 nM, CytD markedly increased both Mf (Fig. 5*A*) and TSA levels (Fig. 5*B*) in IFN-α-treated BMMCs, restoring them to levels comparable to those of control BMMCs. In contrast, 20 nM CytD had no significant effects on the total F-actin, Mf, or TSA/TIA levels of control BMMCs. Beyond structural restoration, treatment with 20 nM CytD also rescued FcεRI-mediated degranulation and TNF-α production in IFN-α-treated BMMCs while having no effect on functional outputs in control cells (Fig. 5*C*).

Similarly, 20 nM CytD restored FcεRI-mediated extracellular Ca^2+^ influx (Fig. 5*D*), the formation of the PLC-γ1-LAT complex, and PLC-γ1 tyrosine phosphorylation (Fig. 5*E*). As expected, however, 20 nM CytD had no effect on these parameters in control BMMCs. Comparable results were observed when BMMCs transfected with siCYP51A1 were treated with 20 nM CytD (Fig. S4). Together, these findings indicate that the disrupted dynamics of the cortical actin cytoskeleton is the ultimate underlying cause of the mast cell-stabilizing effects of IFN-α and CYP51A1 silencing.

### IFN-α alleviates IgE-mediated cutaneous anaphylaxis *via* the CYP51A1-LAN-HMG CoA reductase axis

**Figure 6:**
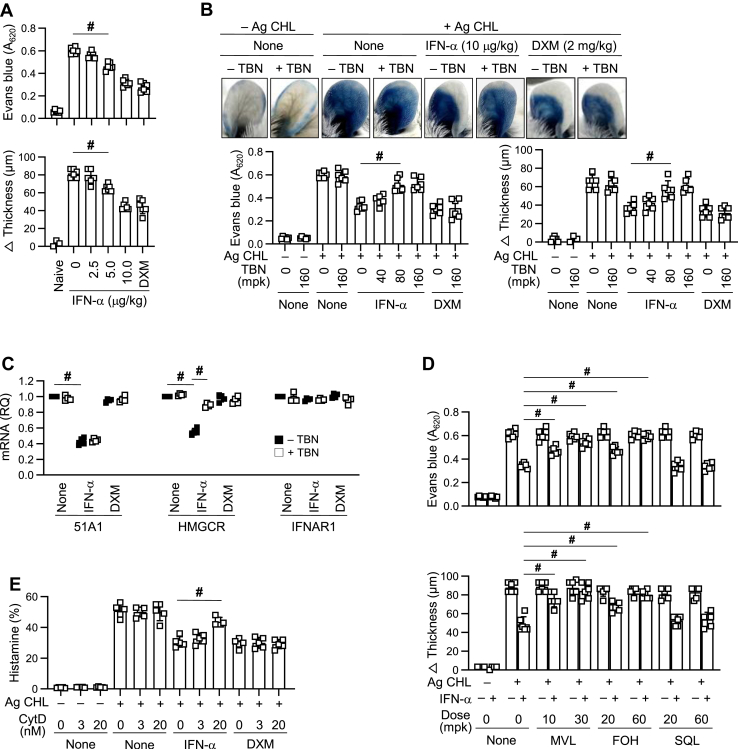
**IFN-α alleviates IgE-mediated cutaneous anaphylaxis through the CYP51A1-lanosterol-HMG CoA reductase axis.***A*, dose-dependent suppression of IgE-mediated cutaneous anaphylaxis by IFN-α. Passive cutaneous anaphylaxis (PCA) assays were performed with mice administered vehicle (none) or IFN-α daily for 2 days (i.p.) at the indicated doses The extent of Evans blue leakage and ear skin thickening is plotted. Dexamethasone (DXM) was administered at 2 mg/kg. *B*, abrogation of IFN-α-suppressed cutaneous anaphylaxis by terbinafine. PCA assays were performed with mice coadministered TBN (i.p., daily for 2 days) at the indicated doses with vehicle (none), IFN-α (10 μg/kg), or DXM (2 mg/kg). Photographs showing the extent of *Evans blue leakage* were taken 60 min after the Ag challenge. *C*, TBN-mediated restoration of IFN-α-suppressed HMGCR mRNA expression, *in vivo*. TBN was coadministered at the indicated doses with vehicle (none), IFN-α or DXM daily for 2 days, followed by FACS-sorting of peritoneal mast cells (PMCs). RNAs isolated from PMCs were subjected to RT-qPCR analyses of the indicated genes. The expression levels relative to those in the vehicle-treated control mice are shown. Data are presented as the mean ± SEM from two separate experiments, each conducted with two mice per group (n = 4). qPCR was performed in triplicate. (# denotes *p* < 0.001 by unpaired Student’s *t* test) *D*, abrogation of IFN-α-suppressed cutaneous anaphylaxis by mevalonate and farnesol. PCA assays were performed with mice coadministered vehicle (none), MVL, FOH, or squalene (SQL) (i.p.) daily for 2 days at the indicated doses with or without IFN-α. *E*, restoration of IFN-α-suppressed FcεRI-mediated histamine release by CytD, *ex vivo*. Total peritoneal exudate cells (PECs) isolated from mice administered vehicle (none), IFN-α, or DXM daily for 2 days were treated *ex vivo* with CytD at the indicated concentration for 20 min prior to challenge ± Ag (n = 5). The results show the mean ± SEM from two separate experiments, each conducted with three mice per group, unless noted otherwise. # and ∗∗ denote *p* < 0.001 and *p* < 0.01, respectively. Statistical significance was determined by one-way ANOVA was performed followed by Tukey’s *post hoc* test. Ag, antigen; CytD, cytochalasin D; FcεRI, high-affinity IgE receptor; FOH, farnesol; HMGCR, HMG-CoA reductase; IFN-α, interferon-α; IgE, immunoglobulin E; MVL, mevalonolactone; RT-qPCR, reverse transcription quantitative PCR; TBN, terbinafine.

Exogenous administration of IFN-α dose-dependently ameliorated anaphylactic symptoms in a mouse model of passive cutaneous anaphylaxis (PCA). Daily administration of IFN-α at 10 μg/kg for two consecutive days reduced the severity of PCA symptoms to levels comparable to those achieved with the maximum dose of dexamethasone (DXM) (2 mg/kg) that did not induce lymphopenia (Fig. 6*A*). Consistent with findings from BMMCs, coadministration of TBN abrogated the ameliorative effect of IFN-α in a dose-dependent manner, while having no impact on symptom severity in sham-treated or DXM-treated mice (Fig. 6*B*).

qRT-PCR analyses of RNA extracted from FACS-sorted peritoneal mast cells (PMCs) revealed that CYP51A1 and HMGCR mRNA expression levels were reduced by approximately 60% and 50%, respectively, in PMCs from IFN-α-treated mice compared with those from sham-treated and DXM-treated mice (Fig. 6*C*). Coadministration of TBN significantly restored HMGCR mRNA expression in PMCs from IFN-α-treated mice, while leaving CYP51A1 expression unaffected. Notably, even at the highest doses tested, IFN-α, TBN, and DXM had no significant effect on the total peritoneal cell counts or on the percentage of c-Kit^+^·FcεRI^+^ PMCs (Fig. S5). These results indicate that the CYP51A1-LAN-HMGCR axis also operate *in vivo* within PMCs, recapitulating the mechanism observed *in vitro* with BMMCs.

MVL and FOH also abrogated the effect of IFN-α in a dose-dependent manner (Fig. 6*D*). At doses of 30 mg/kg and 60 mg/kg, respectively, MVL and FOH nearly completely reversed the ameliorative effect of IFN-α, whereas SQL exerted no significant effect. The counteracting effects of MVL and FOH appeared specific to IFN-α, as neither compound altered symptom severity in sham-treated or DXM-treated mice.

To investigate whether IFN-α suppresses Ag-dependent mast cell activation *in vivo* by stabilizing the cortical actin cytoskeleton, as observed in BMMCs, we conducted an *ex vivo* mast cell degranulation assay. As previously reported (27), when whole peritoneal exudate cells (PECs) isolated from sham-treated control mice—preinjected with anti-DNP IgE for sensitization a few hours before lavage—were challenged *ex vivo* with DNP, more than 50% of total histamine in the PMCs was detected in the supernatants (Fig. 6*E*). This release was markedly reduced when PECs from IFN-α- or DXM-treated mice were challenged. Strikingly, however, histamine release was significantly restored when PECs from IFN-α-treated mice were briefly exposed *ex vivo* to 20 nM CytD before Ag challenge. The counteracting effect of CytD appeared specific to PECs from IFN-α-treated mice, as it had no effect on PECs from sham- or DXM-treated mice. We adopted this whole-PEC degranulation assay (27)—which strictly relies on c-Kit^+^FcεRI^+^ mast cells and Ag-specific IgE sensitization—as part of an effort to facilitate and expedite *ex vivo* mast cell functional assays with untouched mast cells, while also reducing the number of mice required for the study.

## Discussion

The finding that the accumulation of LAN and DHLAN was accompanied by a decrease in HMGCR mRNA expression and an increase in its ubiquitination (Fig. 1) is in line with the report by Chen *et al.* (13). According to their study, LAN and DHLAN differentially regulate HMGCR expression: LAN specifically induced HMGCR degradation by promoting its ubiquitination *via* the INSIG/gp78/VCP complex at the ER, while DHLAN induces both HMGCR degradation and transcriptional suppression. However, since LAN is constantly reduced to DHLAN by 24-dehydrocholesterol reductase (DHCR24) in cells, their respective effects on HMGCR downregulation may ultimately become indistinguishable (Fig. 1*A*).

One might argue that the restoration of bone marrow-derived mast cell (BMMC) functions—which are impaired by IFN-α and CYP51A1 siRNA (Fig. 2)—by TBN is largely due to an off-target effect unrelated to SQLE inhibition. This argument is, however, countered by the observation that TBN did not affect the activation of control BMMCs, treated only with vehicle or transfected with control siRNA. Regarding the proposition that TBN is more effective in IFN-α-treated and siCYP51A1-transfected BMMCs, a plausible explanation is that the accumulation of DHLAN triggers negative feedback regulation of SQLE expression. This process is known to occur in a manner similar to the regulation of HMGCR expression, specifically through the inhibition of SREBP-2 processing (28). This feedback-driven reduction in enzyme concentration effectively increases the inhibitor-to-target ratio, enabling TBN to achieve a significant blockade of LAN synthesis despite its low affinity for the mammalian enzyme; Ki of TBN for mammalian SQLE is over 2000-fold higher than that for its fungal counterpart (29).

Here, it is of note that IFN-I induces the transcriptional upregulation of Cholesterol 25-hydroxylase (Ch25h), which encodes the enzyme catalyzing the hydroxylation reaction of cholesterol at the C25 position (30). The resulting 25-hydroxycholesterol (25HC) interacts with several proteins, including Insig-1, SREBP, and Liver X receptors. Specifically, the interaction of 25HC with Insig-1 and SREBP induces the downregulation of HMC-CoA reductase (HMGCR) through ERAD and transcriptional suppression, respectively (31). Given this literature, it is of great interest that IFN-I utilizes two distinct mechanisms—mediated by LAN- and 25HC—to modulate HMGCR.

Although the relative contribution of each mechanism to the mast cell-stabilizing effect is yet to be elucidated, we postulate that the mechanism of LAN-induced HMGCR downregulation—consequent to transcriptional suppression of CYP51A1— may be the dominant pathway in this context. This postulation is based on our finding that cotreatment with TBN (an inhibitor of SQLE that prevents the accumulation of LAN even when CYP51A1 expression is suppressed) fully restores the mast cells functions impaired by IFN-α. Since TBN specifically mitigates LAN-mediated effects without directly affecting the 25HC-mediated pathway, its ability to rescue the phenotype strongly implicates the CYP51A1-LAN axis as the primary mediator of IFN-α-induced mast cell stabilization. Further studies are required to define the precise interplay between these two pathways in the context of IFN-α-mediated mast cell stabilization.

The finding that cotreatment of IFN-α-treated or siCYP51A1-transfected BMMCs with FOH and GGOH, but not with SQL, restored their activity indicates that the depletion of nonsterol isoprenoids—rather than sterol intermediates—is mainly responsible for the IFN-α-induced suppression of BMMC functions. These results are also consistent with the studies exploring the immunomodulatory effects of various statins (9, 10, 11).

Notably, we have conducted a separate experiment using expression constructs containing the C-terminal farnesylation and geranylgeranylation sequences prenylation sequence from H-Ras and Rac1, respectively, fused to the 3′ end of the GFP gene. Results showed that IFN-α treatment reduced the level of GFP fluorescence in membrane fractions by approximately 30% compared to untreated cells (Naskar *et al.*, unpublished observations). Based on these findings, we anticipate a general decrease in the activity of various small GTPases at the plasma or endosomal membranes to a similar extent. These results are also consistent with the studies exploring the immunomodulatory effects of various statins (9, 10, 11).

In light of the literature and our own findings, it is conceivable that impaired prenylation of Rho-family GTPases (*e.g.*, Cdc42, Rac1, and RhoA) (32)—driven by the depletion of GGPP—underlies the disruption of cortical actin dynamics in BMMCs. Rho-family GTPases play an indispensable role in F-actin reorganization and remodeling (33). This prevents the recruitment of cytosolic signaling proteins like PLC-γ1 to the plasma membrane, thereby disrupting LAT signalosome assembly. The critical role of functional F-actin in PLC-γ1 recruitment and LAT signalosome assembly has been highlighted by studies on mutations in proteins such as WAVE and WASP, which are directly involved in actin reorganization and are regulated by small GTPases (19, 20, 21, 34).

Although the idea that the mast-cell-stabilizing effects of statins and IFN-α stem from the impaired prenylation of small GTPases remains plausible, a caveat arises from our finding that CytD counteracts the effects of both IFN-α and *CYP51A1* silencing by restoring cortical actin dynamics. At the low nanomolar concentrations (<20 nM) utilized in this study, CytD interacts with the barbed ends of F-actin with high specificity. In the mast cell degranulation process, not only Rho-family GTPases but also other small GTPase families—specifically the Rab-family—play a critical role by orchestrating SG trafficking to and fusion with the plasma membrane (35, 36). Unlike Rho-family GTPases, Rab-family GTPases do not rely on F-actin for their function. Instead, their activity is closely associated with microtubules (tubulin). Given that CytD treatment fully restores the level of degranulation suppressed by IFN-α, it is highly probable that Rab-family GTPases remain largely functional despite the decrease in their prenylation after treatment with IFN-α.

Several small biomolecules are also prenylated by FPP and GGPP. For example, ubiquinone and heme are prenylated to form coenzyme Q10 (CoQ10) and heme A, respectively, both of which are key components of the mitochondrial electron transport chain and also play important roles in maintaining cellular redox balance (37, 38). As proper mitochondrial function and redox state in cells are critical for the activation of mast cells (39, 40), future studies investigating the mast cell-stabilizing effects of IFN-α will have to explore how it affects the levels of these small molecules.

The prenylation of Rho-family and Rab-family GTPases is catalyzed by different enzymes with distinct kinetic properties—geranylgeranyltransferase type-I and geranylgeranyltransferase type-II, respectively. Similarly, the prenylation of ubiquinone in the mitochondria and plasma membrane is also mediated by different enzymes, UBIAD1, and CoQ2, respectively (41, 42, 43). Consequently, it is possible that a specific reduction in cellular FPP and GGPP levels may differentially affect the prenylation of various proteins and small biomolecules, depending on the kinetic properties of the respective enzymes. Ongoing studies are examining how IFN-α influence coenxyme Q10 and other prenylated metabolites, and how these alterations impact cortical actin organization and FcεRI-mediated activation of BMMCs.

The observation that CytD counteracted the mast cell-stabilizing effect of IFN-α underscores the importance of diminished dynamics of F-actin as an underlying cause of this mast cell-stabilizing effect (Figs. 5 and S4). In contrast to its dramatic effects on the dynamics of IFN-α-treated and siCYP51A1-transfected BMMCs, CytD had no significant impact on F-actin remodeling in control BMMCs. Although the selective effects of CytD on IFN-α-treated and siCYP51A1-transfected BMMCs are not fully elucidated, we postulate that control BMMCs remain unresponsive because their cortical actin already undergoes rapid turnover. Thus, the subtle alteration in the remodeling rate caused by barbed-end binding of CytD at such a low concentration may not noticeably affect the overall dynamics of the cortical actin cytoskeleton in these cells.

In contrast, under conditions where F-actin remodeling is severely impaired, as observed in IFN-α-treated BMMCs, the displacement of specific actin-binding proteins from the barbed-ends by CytD may trigger a structural reorganization in F-actin fibers. We speculate that this targeted perturbation in an otherwise stalled system is sufficient to reinvigorate actin dynamics to a measurable extent. Future studies aimed at better understanding the selective effect of CytD on IFN-α-treated cells should investigate how IFN-α or CYP51A1 silencing modifies the activity of barbed end-binding proteins—including Ena/VASP, formins, or capping proteins—while not excluding other F-actin binding proteins (24, 26, 44).

Since IFNAR is ubiquitously expressed, IFN-α-induced impairment of cortical actin dynamics—mediated through the CYP51A1-LAN-HMGCR axis—likely influences a broad range of immune and nonimmune cells, albeit to a varying degree depending on its expression level. Notably, however, this mechanism appears particularly effective in suppressing mast cell activation compared to other cell types, even within the immune compartment. According to a study by Ye *et al.*, inotodiol (22-hydroxylanosterol) (INO), a fungal metabolite of LAN, most potently inhibits FcεRI-mediated mast cell activation relative to other immune responses, including Ag-specific B and T cell activation, as well as LPS-induced macrophage and neutrophil activation (45). Recently, we found that INO also exerts its mast cell-stabilizing effects primarily through impairment of cortical actin dynamics, resulting from HMGCR downregulation *via* ubiquitin-mediated ERAD (46).

Although the precise reason why mast cell activation is particularly susceptible to HMGCR downregulation remained be unraveled, we speculate that the unique architecture of the mast cell cortical actin cytoskeleton may contribute to this sensitivity. Although cortical F-actin formation typically intensifies upon receptor triggering in other immune cells, such as T cells (47), the density of cortical F-actin in mast cells is already high in the resting state. This dense network serves as a physical barrier that prevents the fusion of SGs with the plasma membrane (48, 49, 50). Upon FcεRI triggering, the cortical F-actin network promptly clears from the cell periphery, allowing SGs to migrate toward and fuse with the plasma membrane (51, 52). Thus, the stabilization of cortical F-actin, which renders the network more rigid and less dynamic, may have a more profound inhibitory effect on receptor-triggered signal propagation in mast cells than in other cell types by spatially interfering with the recruitment of cytosolic signaling proteins to the plasma membrane.

Considering the pharmacokinetic properties of IFN-α in mice (53, 54, 55), the peak blood concentration following an intraperitoneal injection of 10 μg/kg (∼250 ng/mouse) likely reaches ∼5 ng/ml and declines to sub ng/ml levels within 3 h. Given these kinetics, the finding that IFN-α—administered daily for 2 days at this dose—alleviated anaphylaxis symptoms by over 50% is intriguing. This is especially notable because a 2-day IFN-α treatment *in vitro*, even at 25 ng/ml, had a minimal effect on SG release and TNF-α production in BMMCs. Conceivably, tissue-resident mast cells are programed to respond more sensitively to changes in IFN-I concentration than *in vitro*-differentiated BMMCs. Despite the apparent disparity in efficacy between the *in vitro* and *in vivo* settings, our findings clearly indicate that IFN-α stabilizes mast cells through a common mechanism in both environments (Fig. 6).

**Figure 7:**
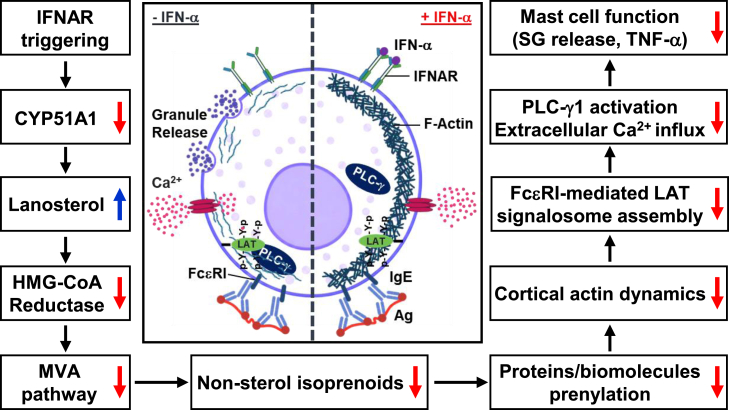
**Schematic representation of the proposed mechanism for the mast cell stabilizing effects of IFN-α.** IFN-α induces transcriptional suppression of the CYP51A1 gene in mast cells. This attenuates the conversion of lanosterol to downstream intermediates in the cholesterol biosynthesis pathway. Consequently, lanosterol accumulates, triggering both SREBP-mediated transcriptional suppression and ERAD-mediated degradation of HMGCR. This HMGCR downregulation leads to the blockade of the MVA pathway, which is responsible for the synthesis of FPP and GGPP required for prenylation of various proteins and small biomolecules. The shortage of these nonsterol isoprenoids results in the loss of cortical actin dynamics *via* a mechanism that warrant further investigation. This lack of dynamic actin rearrangement obstructs recruitment of PLC-γ1 to LAT, thereby preventing the assembly of multiprotein signaling complex essential for PLC-γ1 activation and the subsequent extracellular influx of Ca^2+^. ERAD, the endoplasmic reticulum-associated degradation; FPP, farnesyl pyrophosphate; GGPP, geranylgeranyl pyrophosphate; HMGCR, HMG-CoA reductase; IFN-α, interferon-α; MVA, mevalonate; LAT, linker for activation of T cell; PLC-γ1, phospholipase C-γ1; SREBP, sterol regulatory element-binding protein.

Taken together, we propose a working model for the mast cell-stabilizing and anti-anaphylactic effects of IFN-α as follows (Fig. 7): (i) IFN-α inhibits the MVA pathway through the CYP51A1-LAN-HMGCR axis in mast cells. (ii) This reduces the availability of FPP and GGPP, hindering the prenylation of small GTPases and/or other low-molecular-weight biomolecules. (iii) As a result, the dynamics of the cortical actin cytoskeleton are disrupted, obstructing the recruitment of cytosolic signaling proteins (*e.g.*, PLC-γ1) to the plasma membrane and the subsequent assembly of the LAT signalosome. (iv) This prevents the activation of store-operated calcium entry evoked by FcεRI triggering, which is a prerequisite for both SG release (degranulation) and the downstream signal propagation for TNF-α gene transcription (56, 57).

## Experimental procedures

### Reagents

The sources of all reagents used in this study are specified in the Supplementary Materials. Inotodidol, used as an internal standard in the HPLC analyses, was purified from the Chaga mushroom (*Inonotus obliquus*) to a purity of over 97% as previously described (58).

### Animals

BALB/C and C57BL/6 mice (6–10 weeks old, male) were purchased from SAMTAKO Bio Korea. IFNAR-deficient mice in B6 background, a kind gift from Dr Bumseok Kim at the College of Veterinary Medicine, Jeonbuk National University (59), were bred in an SPF condition at the Core Animal Facility, Chungnam National University (CNU). The animal experiment protocols were approved by the Institutional Animal Care and Use Committee (IACUC) in CNU (CNU-00996), and all animal experiments were carried out per the approved protocols.

### *In vitro* differentiation of BMMCs

Bone marrow isolated from the tibia and femur was cultured in RPMI medium (10% FBS, 1% Penicillin-Streptomycin-L-Glutamine, 10 mM Hepes, and 50 μM 2-mercaptoethanol) supplemented with IL-3 (10 ng/ml) for 4 to 6 weeks (60).

### Silencing of CYP51A1 gene expression

siRNA specific for CYP51A1 (sc-1429) and the control siRNA (sc-44230) were purchased from Santa Cruz Biotechnology. BMMCs were transfected using the Neon Transfection System (Thermo Fisher Scientific) at a single pulse of 2000 V for 20 ms, according to the manufacturer’s instructions.

### RT-qPCR

Total RNAs and cDNAs were prepared using the AccuPrep Universal RNA Extraction Kit and AccuPower RT/PCR PreMix, respectively (Bioneer). qPCR was performed in triplicate using SYBR Green mix on AriaMx real time PCR system (Agilent), and the relative gene expression levels of CYP51A1, HMGCR, and IFNAR mRNAs to β-actin were computed with the 2ˆ − ΔΔCt method (61). β-actin forward: 5′-GAAGTGTGACATCCG-3′, reverse: 5 -TGCTGATCCACATCTGCTGGA-3′; CYP51A1 forward: 5′-CTGCCCGCTGGAGCGAAAAG-3′, reverse: 5 -CACAGGTGTTGTCAGCCGACC-3′; HMGCR forward: 5 -CCTGTAACTCAGAGGGTCAAG-3′, reverse: 5 -CCAGCGACTGTGAGCATGAA -3′; IFNAR1 forward: 5′-AGCCACGGAGAGTCAATGG-3′, reverse: 5′-GCTATGACACGAAACTGTGTTTT-3′.

### IgE-mediated degranulation and TNF-α production assays

BMMCs (2 × 10^5^ cells) sensitized with anti-DNP IgE (1 μg/ml) overnight were washed, and challenged with DNP-BSA (100 ng/ml) at 37 °C in Tyrode’s A buffer (135 mM NaCl, 5 mM KCl, 20 mM Hepes, 5.6 mM glucose, 1 mM MgCl_2_, 1.8 mM CaCl_2_, 0.05% bovine serum albumin, pH 7.4). For the degranulation assay, 20 min after Ag challenge, BMMCs in a 96-well plate were centrifuged for 1 min at 450 × g, and the activities of β-hexosaminidase in the supernatant and the cell lysate were measured separately. The extent of degranulation (% degranulation) was calculated as described (62). For the TNF-α production assay, ELISA was conducted using the supernatants collected 60 min after the Ag challenge.

### Flow cytometry Ca^2+^ assay

BMMCs (5 × 10^5^), sensitized with anti-DNP IgE overnight, were loaded with Fluo-3 AM ester for 45 min at 37 °C and resuspended in a calcium assay buffer (1× HBSS, 1 mM MgCl_2_, 0.5% BSA) with or without 1 mM CaCl_2_ after thorough washing. Baseline fluorescence (Ex/Em = 488/525 nm) was measured for 20 s in a flow cytometer (FACS Aria Fusion, BD Biosciences) set at 37 °C before the addition of DNP-BSA (63). Changes in intracellular Ca^2+^ levels were monitored over 140 s.

### Western blot analyses

BMMCs (2 × 10^6^), sensitized with anti-DNP IgE (1 μg/ml) overnight, were challenged with DNP-BSA (100 ng/ml) for 30 s at 37 °C, and lysed in RIPA buffer (50 mM Tris-HCl, pH 8.0, 150 mM NaCl, 2 mM MgCl_2_, 1.5% NP-40, 0.1% SDS, and 0.5% sodium deoxycholate) supplemented with a protease/phosphatase inhibitor cocktail for 15 min on ice. The cell lysates were centrifuged at 17,000 × *g* for 15 min to remove the debris, and the cleared lysates were subjected to Western blot analyses. West Femto Maximum Sensitivity Substrate (Thermo Fisher Scientific) was used as an HRP substrate, and chemiluminescence was detected using iBright CL1500 Imaging System (Thermo Fisher Scientific).

### HMGCR immunoblotting and immunoprecipitation

BMMCs (3 × 10^6^) were lysed in RIPA buffer supplemented with a protease inhibitor cocktail. The protein concentrations of lysates were measured using the BCA method. The cell lysates were then mixed with an equal volume of membrane protein solubilization buffer (62.5 mM Tris-HCl, pH 6.8, 15% SDS, 8 M urea, 10% glycerol, and 100 mM DTT) plus 4 x gel loading buffer (150 mM Tris-HCl, pH 6.8, 12% SDS, 30% glycerol, 6% 2-mercaptoethanol, and 0.02% bromophenol blue), followed by incubation at 37 °C for 30 min. The resulting samples were subjected to Western blot analyses using anti-HMGCR mAb.

For the HMGCR ubiquitination assay, BMMCs were lysed in immunoprecipitation buffer (1 × PBS, 1% Triton X-100, 5 mM EDTA, and 5 mM EGTA) supplemented with a protease inhibitor cocktail. Lysates were precleared with Protein A/G Plus-Agarose (sc-2003) (Santa Cruz Biotechnology) for 1 h at 4 °C. The precleared cell lysates were incubated with rabbit polyclonal anti-HMGCR Ab plus Protein A/G Plus-Agarose overnight at 4 °C. After thorough washing, the beads were incubated at 95 °C for 10 min in 2 x gel-loading buffer and then spun down. The supernatant was then mixed with an equal volume of membrane protein solubilization buffer and incubated at 37 °C for 30 min before immunoblotting with an anti-ubiquitin mAb (64).

### Measurement of Triton X-100-soluble and insoluble actin

Triton X-100-soluble and insoluble actin were fractionated as described previously, with modifications (65, 66, 67). BMMCs (2 × 10^6^) were incubated for 30 s on ice in 100 μl of the cytoskeleton buffer (actin stabilization buffer) (10 mM PIPES, pH 6.8, 100 mM NaCl, 1 mM EGTA, 1 mM ATP, 3 mM MgCl_2_, and 300 mM sucrose) containing 0.25% Triton X-100, followed by centrifugation at 450 × *g* for 1 min in a 96-well plate. Then, the supernatant was saved in a separate tube. The pellet was washed once with the same buffer, and the resulting supernatant was combined with the first. The cell pellet was re-suspended in the SDS-PAGE gel-loading buffer and boiled for 5 min for Western blot analyses.

Alternatively, the cell pellet was resuspended in 1× PBS with 4% paraformaldehyde and incubated for 20 min at r. t., followed by staining with iFluor 488-conjugated phalloidin (ab176753) (Abcam) for flow cytometry (68).

### Fluorescence recovery after photobleaching assay

A Lifeact-GFP expression construct was prepared using the Lifeact-mCherry-expression construct, which was a gift from Michael Davidson (Addgene plasmid # 54491; http://n2t.net/addgene:54491; RRID:Addgene_54491), by replacing the mCherry coding region with GFP coding sequence in frame. BMMCs transfected with the Lifeact-GFP expression construct were seeded in a 6-well glass bottom plate coated with Cell-Tak. Twenty-four hrs after transfection, the plate was placed on a confocal microscope, and ROIs were selected for photobleaching. Photobleaching was conducted with maximum laser power for 5 s. Following photobleaching, the extent of fluorescence recovery was monitored by capturing live images over a 120-s period (69). Images were collected using a Zeiss LSM 880 confocal laser scanning microscope equipped with a 63 x oil immersion objective lens. Microscope image processing was performed with ImageJ software, and the data analyses were conducted with Zen software (Zeiss).

### HPLC analyses for quantifying LAN and DHLAN

HPLC analyses of LAN and DHLAN was performed as described by others with modifications (70, 71). Briefly, BMMCs (5 × 10^6^ cells) were washed twice with ice-cold PBS and lysed with 10% (w/v) KOH. Inotodiol (INO), used as an internal standard, was spiked to the cell lysate to 50 ng/ml. Then, the cell lysates were treated sequentially with chloroform-methanol (2:1, v/v) and distilled water to obtain the lipid and water-soluble fractions. The lipid fractions were further fractionated into the saponifiable and non-saponifiable fractions using hexane. Nonsaponifiable lipids in hexane fraction were used for HPLC analyses (12).

HPLC analyses was conducted using an YL9100 HPLC system (Youngin), equipped with a Sunfire C18 column (4.6 mm × 150 mm, 5 μm particle size, Waters). The chromatographic separation was carried out under the following conditions: the mobile phase consisted of a mixture of acetonitrile and water (H_2_O), starting at a volume ratio of 70:30 (acetonitrile/H_2_O) at 0 min, and changing linearly to 95:5 at 30 min. The flow rate was set at 1 ml/min. UV detection was employed at a wavelength of 254 nm. The major peak representing cholesterol, which accounts for approximately 95% of the total sterols, is concealed using the built-in Clarity Chromatography software to clarify the HPLC profiles and ensure accurate quantification of LAN and DHLAN.

Mass spectra were acquired using a Synapt G2-HDMS mass spectrometer (Waters), controlled by MassLynx 4.1 software.

### PCA assay

Mice were injected with anti-DNP IgE (3 μg) or PBS under the ear skin. The following day, they were injected (i.v.) with DNP-BSA (80 μg) plus Evans blue (2.5 mg). 60 min after the injection, the mice were euthanized, and the thickness of the ear pinnae was measured with a caliper. Evans blue dye extravasated into the ear skin was extracted overnight in 500 μl of DMSO, and the absorbance of the solution was measured at 620 nm (72).

### *Ex vivo* mast cell degranulation assay

PECs were collected *via* lavage from mice that were injected (i.p.) with anti-DNP IgE (3 μg) 1 day prior and then washed in Tyrode’s B buffer (137 mM NaCl, 12 mM NaHCO_3_, 2.7 mM NaHPO_4_, 5.6 mM glucose, and 0.1% gelatin). The PECs (5 × 10^5^) were resuspended in Tyrode’s A buffer (130 mM NaCl, 10 mM Hepes, 1 mM MgCl2, 5 mM KCl, 1.4 mM CaCl2, 5.6 mM glucose, and 1% BSA) and challenged *ex vivo* with DNP-BSA (5 ng/ml) for 10 min at 37 °C. Histamine released into the supernatant and retained within the cells was measured using ELISA. The proportion of secreted histamine relative to the total was then calculated (27).

### Statistical analyses

Statistical analyses were conducted using GraphPad Prism 6 (GraphPad Software). The significance of the differences among multiple groups was assessed using nonparametric one-way ANOVA, followed by Tukey’s multiple comparison test for *post hoc* analyses. A *p*-value less than 0.05 was considered statistically significant.

## Data availability

All data supporting the findings of this study are available within the article and its supporting information. The data that support the findings of this study are available from the corresponding author upon reasonable request.

## Supporting information

This article contains supporting information.

## Conflict of interest

The authors declare that they have no conflicts of interest with the contents of this article.
