# Supplementary material for: Impaired cortical actin dynamics via lanosterol-dependent HMG-CoA reductase downregulation mediates IFN-α-induced mast cell stabilization
Source: J Biol Chem. 2026 May 16;302(7):113161. doi: 10.1016/j.jbc.2026.113161 (PMC13279192; doi:10.1016/j.jbc.2026.113161)
Supplement: Supplementary Data [file mmc6.docx]

**Materials**

| Reagent | Source | Identifier |
| --- | --- | --- |
| IFN-α | BioLegend | 752804 |
| Anti-Phosphotyrosine (pY20) mAb | ThermoFisher Scientific | 03-7700 |
| Anti-phospho-LAT (Y191) mAb | Abcam | Ab314040 |
| Anti-phospho-PLCγ1 (Tyr783) mAb | BioLegend | 612402 |
| Anti-HMGCR (C-1) mAb | Santa Cruz Biotechnology | sc-271595 |
| Anti-Ubiquitin (P4D1) mAb | Cell Signaling Technology | #3936 |
| Anti-LAT (E3U6J) mAb | Cell Signaling Technology | #45533 |
| Anti-FcR γ chain (E6Y1A) mAb | Cell Signaling Technology | #78401 |
| Anti-PLCγ1 mAb | Cell Signaling Technology | #2822s |
| Anti-Phospho Syk  (Tyr323) mAb | Cell Signaling Technology | #2715 |
| Anti-Syk mAb | Cell Signaling Technology | #2712 |
| Anti-Phospho Lyn (Tyr507) mAb | Cell Signaling Technology | #2731 |
| Anti-Lyn mAb | Cell Signaling Technology | #2732 |
| Anti-DNP IgE | Sigma-Aldrich | D8406 |
| Anti-β-actin Antibody (C4) mAb | Santa Cruz Biotechnology | sc-47778 |
| Anti-SLP76 mAb | Santa Cruz Biotechnology | sc-13151 |
| Anti-SREBP2 mAb (1C6) | Santa Cruz Biotechnology | sc-13552 |
| Lanosterol | Sigma-Aldrich | L5768 |
| Dihydrolanosterol | Sigma-Aldrich | 700067P |
| Evans blue | Sigma-Aldrich | E2129 |
| Mevalonolactone | Sigma-Aldrich | M4667 |
| Farnesol | Sigma-Aldrich | F203 |
| Geranylgeraniol | Sigma-Aldrich | G3278 |
| Squalene | Sigma-Aldrich | S3626 |
| BSA | Sigma-Aldrich | A7030 |
| HEPES | Sigma-Aldrich | H3375 |
| PIPES | Sigma-Aldrich | P1851 |
| NaCl | Sigma-Aldrich | S9888 |
| MgCl_2_ | Sigma-Aldrich | [M4880](https://www.sigmaaldrich.com/KR/ko/product/sigma/m4880) |
| Potassium hydroxide | Merck-Millipore | [484016](https://www.sigmaaldrich.com/KR/ko/product/sigald/484016) |
| Tween 20 | Sigma-Aldrich | P1379 |
| 2-Mercaptoethanol | Sigma-Aldrich | M6250 |
| *p*-nitrophenyl N-acetyl-β-D-glucosaminide (PNAG) | Sigma-Aldrich | N9376 |
| TMB solution | Sigma-Aldrich | T0440 |
| Horse serum | Sigma-Aldrich | H1270 |
| DNase-Free RNase | Sigma-Aldrich | 11119915001 |
| Paraformaldehyde | Sigma-Aldrich | P6148 |
| Trition X-100 | Sigma-Aldrich | T8787 |
| Propidium iodide (PI) | Sigma-Aldrich | 537059 |
| Protease inhibitor cocktail | Sigma-Aldrich | P8340 |
| Phosphatase inhibitor cocktail | Sigma-Aldrich | P0044 |
| RPMI 1640 medium | ThermoFisher Scientific | 11875119 |
| Fetal bovine serum (FBS) | ThermoFisher Scientific | [16140071](https://www.thermofisher.com/order/catalog/product/16140071) |
| Tris Base | Sigma-Aldrich | 252859 |
| Potassium chloride | Merck Millipore | 104936 |
| Penicillin-streptomycin-glutamine solution | ThermoFisher Scientific | 10378016 |
| DNP-BSA | ThermoFisher Scientific | A23018 |
| RBC lysis buffer | ThermoFisher Scientific | 00-4333-57 |
| Fluo-3 | ThermoFisher Scientific | F1242 |
| SuperSignal™ West Femto Maximum Sensitivity Substrate | ThermoFisher Scientific | [34577](https://www.thermofisher.com/order/catalog/product/34577) |
| EDTA | Biofact | PB131-500 |
| Bromophenol Blue | Merck-Millipore | B5525 |
| Glucose | Sigma-Aldrich | 45-G8270 |
| Calcium chloride | Merck-Millipore | 102378 |
| Urea | Merck Millipore | [U5378](https://www.sigmaaldrich.com/KR/ko/product/sigma/u5378) |
| Glycine | Sigma-Aldrich | G7126 |
| SDS | Sigma-Aldrich | L3771 |
| Sodium deoxycholate | Sigma-Aldrich | D6750 |
| Glycerol | Sigma-Aldrich | G5516 |
| DTT | Sigma-Aldrich | D0632 |
| SYBR Green mix | Biofact | DQ384 |
| Cytochalasin D | Sigma-Aldrich | C8273 |
| Protein A/G PLUS-Agarose | Santa Cruz Biotechnology | sc-2003 |
| Horseradish peroxidase (HRP)-conjugated streptavidin | BioLegend | 405210 |
| Phalloidin (iFluor 488-conjugated) | Abcam | ab176753 |
| rmIL-3 (carrier-free) | BioLegend | 578006 |
| Cell-Tak | Corning | 354240 |
| AccuPrep® Universal RNA Extraction Kit | Bioneer | K-3140 |
| AccuPower® RT/PCR PreMix | Bioneer | K-2044 |
| TNF-α ELISA kits | BD Biosciences | BD 555268 |
| Histamine ELISA kits | Abcam | Ab213975 |
| GFP-expression plasmid | Sino Biological | CV026 |
| mCherry-Lifeact-7 expression plasmid | Addgene | #54491 |
