# Supplementary material for: Impaired cortical actin dynamics via lanosterol-dependent HMG-CoA reductase downregulation mediates IFN-α-induced mast cell stabilization
Source: J Biol Chem. 2026 May 16;302(7):113161. doi: 10.1016/j.jbc.2026.113161 (PMC13279192; doi:10.1016/j.jbc.2026.113161)
Supplement: Supplementary Figures [file mmc7.pdf]

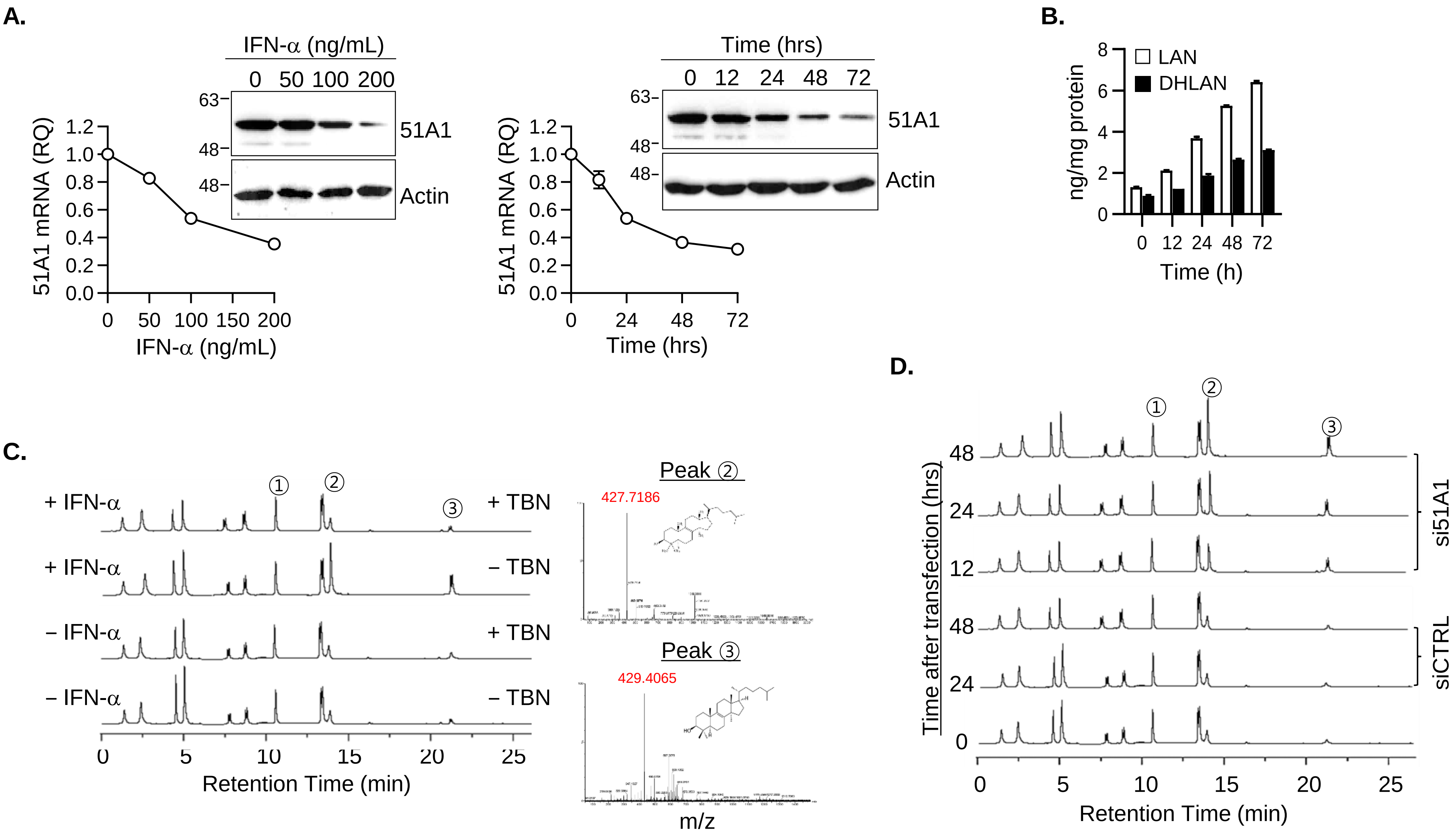
A.
IFN-α (ng/mL)
0 50 100 200
63
48
51A1
Actin
51A1 mRNA (RQ)
1.2 1.0 0.8 0.6 0.4 0.2 0.0
0 50 100 150 200
IFN-α (ng/mL)
Time (hrs)
0 12 24 48 72
63
48
51A1
Actin
51A1 mRNA (RQ)
0 24 48 72
Time (hrs)
B.
ng/mg protein
8 6 4 2 0
LAN
DHLAN
0 12 24 48 72
Time (h)
C.
+ IFN-α + TBN
+ IFN-α − TBN
− IFN-α + TBN
− IFN-α − TBN
① ② ③
0 5 10 15 20 25
Retention Time (min)
Peak ②
427.7186
Peak ③
429.4065
m/z
D.
① ② ③
Time after transfection (hrs)
48 24 12 48 24 0
si51A1
siCTRL
0 5 10 15 20 25
Retention Time (min)

Figure S1

Figure S1. IFN-α and siCYP51A1 downregulate HMGCR by inducing LAN accumulation.

(A)IFN-α-induced downregulation of CYP51A1 mRNA and protein expression. RNAs and lysates prepared from BMMCs, treated with IFN-α at varying concentrations for 48 h (left) or at 200 ng/mL for different durations (right), were subjected to RT-qPCR and immunoblotting, respectively. RQ stands for "relative quantification". Note that the actin IB shown here is the same as in Figure 1B; HMGCR, CYP51A1, and actin were detected by dividing a single membrane into three strips based on their respective molecular weights.

(B) IFN-α-induced accumulation of LAN and DHLAN. Nonsaponifiable lipids were extracted from BMMCs treated with IFN-α (200 ng/mL) for the indicated durations and subjected to HPLC analysis. The graph shows cellular levels of LAN and DHLAN normalized to total protein in the cell lysates (mg). The data are presented as the mean ± SD of one representative experiment from three independent repeats.

(C) The counteracting effect of TBN on IFN-α-induced accumulation of LAN and DHLAN. BMMCs were co-treated with vehicle (DMSO) or TBN (20 μM) for the final 10 h of the 48-h incubation in the presence or absence of IFN-α, followed by lipid extraction and HPLC analysis. Peaks corresponding to LAN (②) and DHLAN (③) are shown, alongside inotodiol (INO, ①) used as an internal control. Mass profiles for peak ② and ③ are provided, matching the theoretical masses of LAN and DHLAN, respectively. (Note: The major peak for cholesterol, representing ~95% of the total sterols, was omitted using the built-in Clarity Chromatography software to enhance the visibility and quantification of LAN and DHLAN.)

(D) CYP51A1 silencing-induced accumulation of LAN and DHLAN. BMMCs transfected with siCYP51A1 or siCTLR (control siRNA) were cultured for the indicated time periods, followed by lipid extraction and for HPLC analysis.

Figure S1 (Cont'd)

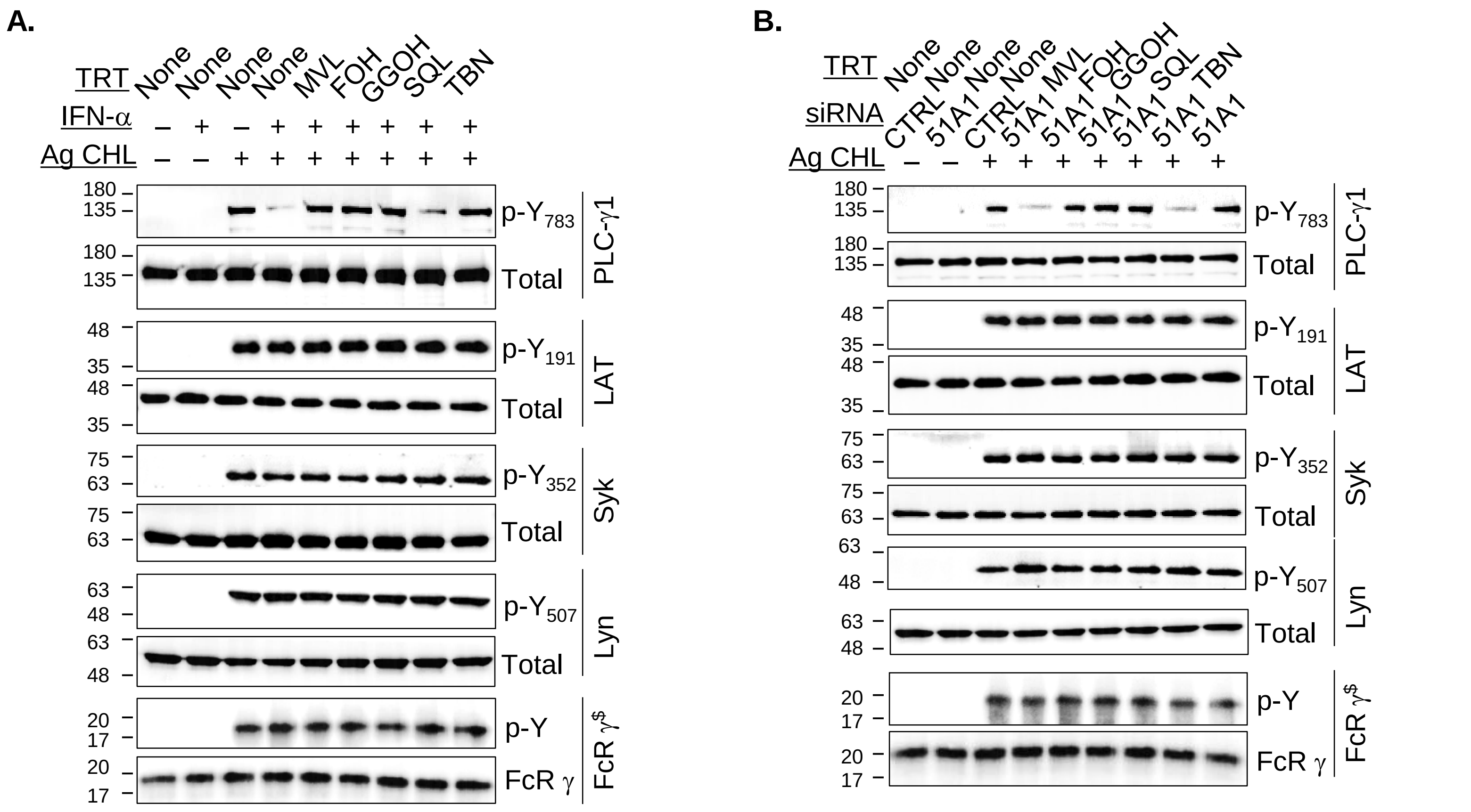

Figure S2. IFN-α and CYP51A1 silencing inhibit FcεRI-mediated PLC-γ1 activation without affecting upstream signaling events.

BMMCs were sensitized with IgE overnight and treated with MVL (30 μM), FOH (30 μM), GGOH (30 μM), SQL (90 μM), or TBN (20 μM) for the final 10 h during a 48-h IFN-α (200 ng/mL) treatment (A) or a 24-h culture following transfection with siRNA (B). Cells were then challenged with or without antigen in a Ca^{2+}-free buffer for 30 s, followed by lysis for immunoblotting.

($ indicates the cell lysate was IPed with an anti-FcR γ-chain mAb, followed by WB analysis.)

Figure S2

A.

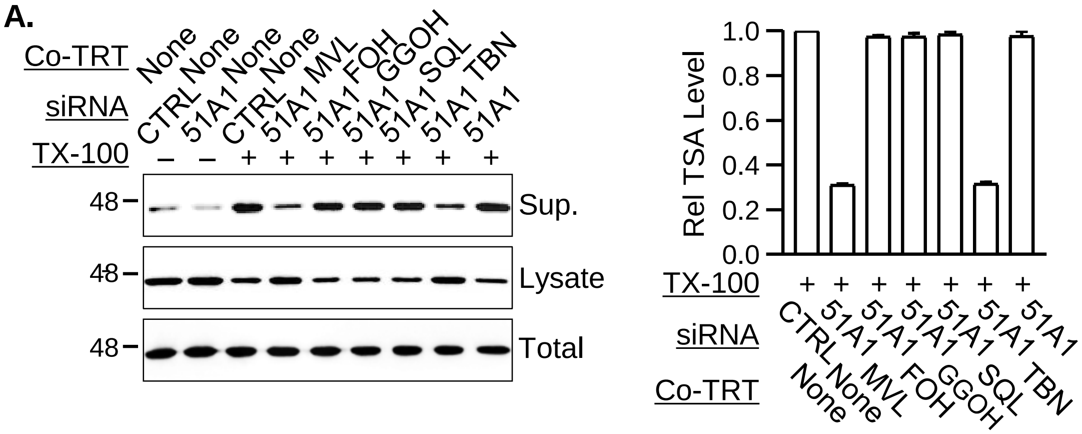
Co-TRT
siRNA
TX-100
None
CTRL
None
51A1
None
CTRL
None
51A1
MVL
51A1
FOH
51A1
GGOH
51A1
SQL
51A1
TBN
51A1
48
Sup.
Lysate
Total
Rel TSA Level
1.0
0.8
0.6
0.4
0.2
0.0

B.

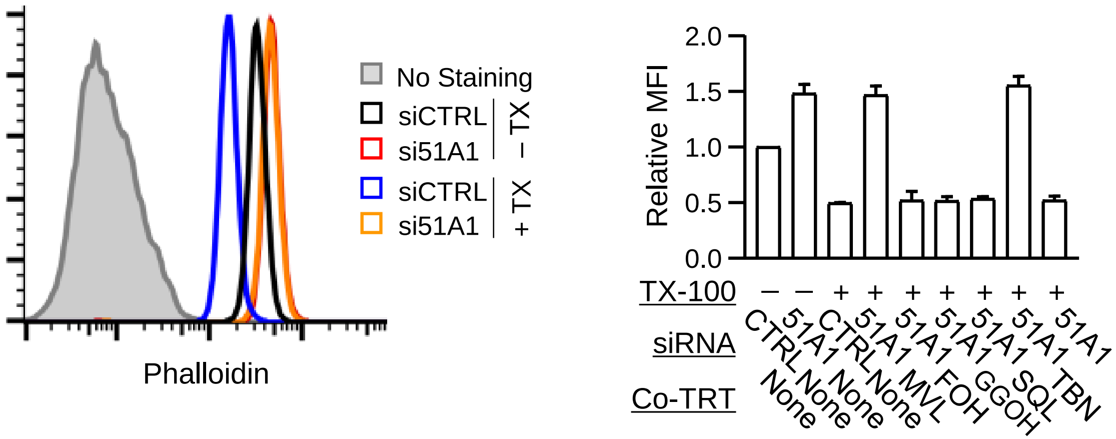
No Staining
siCTRL
si51A1
– TX
siCTRL
si51A1
+ TX
Phalloidin
Relative MFI
2.0
1.5
1.0
0.5
0.0
TX-100
siRNA
Co-TRT

Figure S3

Figure S3. CYP51A1 silencing impairs cortical actin dynamics via the CYP51A1-LAN-HMGCR axis.

(**A**) Restoration of TSA reduced by siCYP51A1 via TBN and MVA pathway intermediates. BMMCs, treated with TBN or a MVA pathway intermediate for the last 10 h of a 24-h incubation post-transfection with the siRNAs, were washed with an actin-stabilizing buffer ± Triton X-100. Supernatants and cell lysates were subjected to immunoblotting with an anti-actin mAb as means to quantify Triton X-100-soluble actin (TSA) and Triton X-100-insoluble actin (TIA), respectively. Actin band intensities of the supernatants were normalized to the total (supernatant + cell lysate), and the relative changes compared to the control are shown. The data present the mean ± SD of three independent experiments.

(**B**) Restoration of TIA elevated by IFN-α via TBN and MVA pathway intermediates. BMMCs, treated and washed as described above, were fixed with PFA and stained with fluorescently labeled phalloidin for flow cytometry analyses. Representative histograms are shown. The mean fluorescence intensities (MFIs), relative to those of control BMMCs, are also presented as the mean ± SD from three experiments performed in duplicate.

Figure S3 (Cont’d)

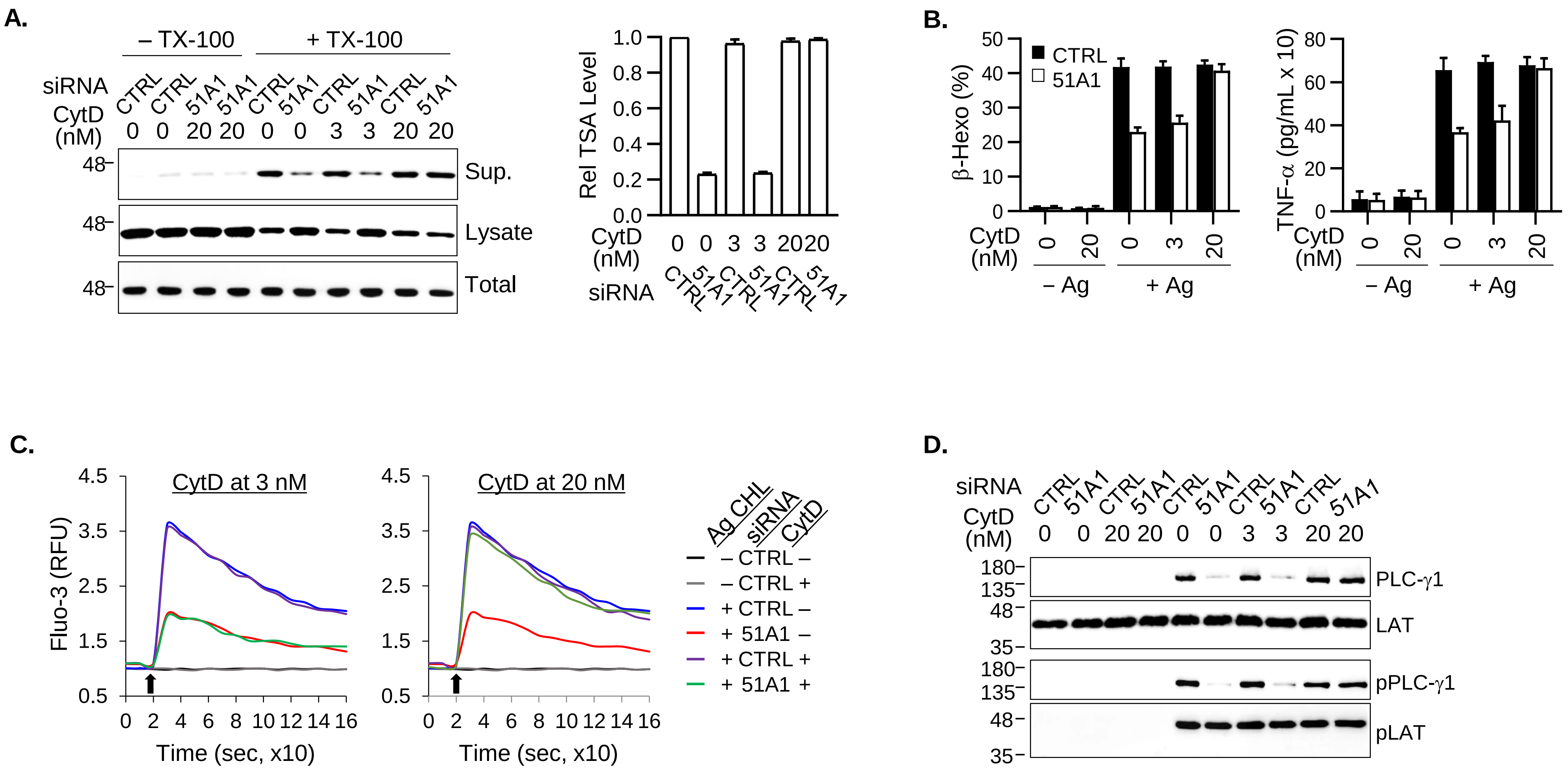
A.
– TX-100
+ TX-100
siRNA
CytD (nM)
CTRL CTRL 51A1 51A1 CTRL 51A1 CTRL 51A1 CTRL 51A1
0 0 20 20 0 0 3 3 20 20
48
Sup.
Lysate
Total
Rel TSA Level
1.0 0.8 0.6 0.4 0.2 0.0
B.
β-Hexo (%)
CTRL
51A1
– Ag
+ Ag
TNF-α (pg/mL x 10)
C.
CytD at 3 nM
CytD at 20 nM
Fluo-3 (RFU)
Time (sec, x10)
Ag CHL siRNA CytD
– CTRL –
– CTRL +
+ CTRL –
+ 51A1 –
+ CTRL +
+ 51A1 +
D.
180
135
35
PLC-γ1
LAT
pPLC-γ1
pLAT

Figure S4

Figure S4. CtyD counteracts the mast cell-stabilizing effect of CYP51A1 silencing by restoring cortical actin dynamics.

(A) Normalization of siCYP51A1-induced changes in TSA and TIA levels by CtyD. BMMCs, treated with CtyD (3 or 20 nM, 20 min) for the final 20 min during a 24-h culture post-transfection with siCYP51A1 or siCTRL. Cells were washed with actin stabilization buffer ± Triton X-100. Supernatants and cell lysates were subjected to immunoblotting with an anti-actin mAb as means to quantify Triton X-100-soluble actin (TSA) and Triton X-100-insoluble actin (TIA), respectively. Band intensities of the supernatants were normalized to the total (supernatant + cell lysate), and the relative changes compared to the control are also plotted.

(B) Restoration of FcεRI-mediated degranulation and TNF-α production impaired by CYP51A1 silencing using CtyD. BMMCs, sensitized overnight with IgE during a 24-h culture post-transfection with siRNA, were treated with CtyD for 20 min before challenge with or without Ag.

(C) Restoration of FcεRI-mediated extracellular Ca^{2+} influx impaired by CYP51A1 silencing using CtyD. BMMCs, sensitized and treated as described above, were subjected to the Ca^{2+} assay in a Ca^{2+}-containing buffer.

(D) Restoration of FcεRI-mediated LAT-PLC-γ1 complex formation impaired by CYP51A1 silencing using CtyD. BMMCs, sensitized and treated as described above, were challenged with or without Ag in a Ca^{2+}-free buffer. Cell lysates were subjected to immunoprecipitation with an anti-LAT mAb, followed by immunoblotting using the indicated Abs.

Figure S4 (Cont'd)

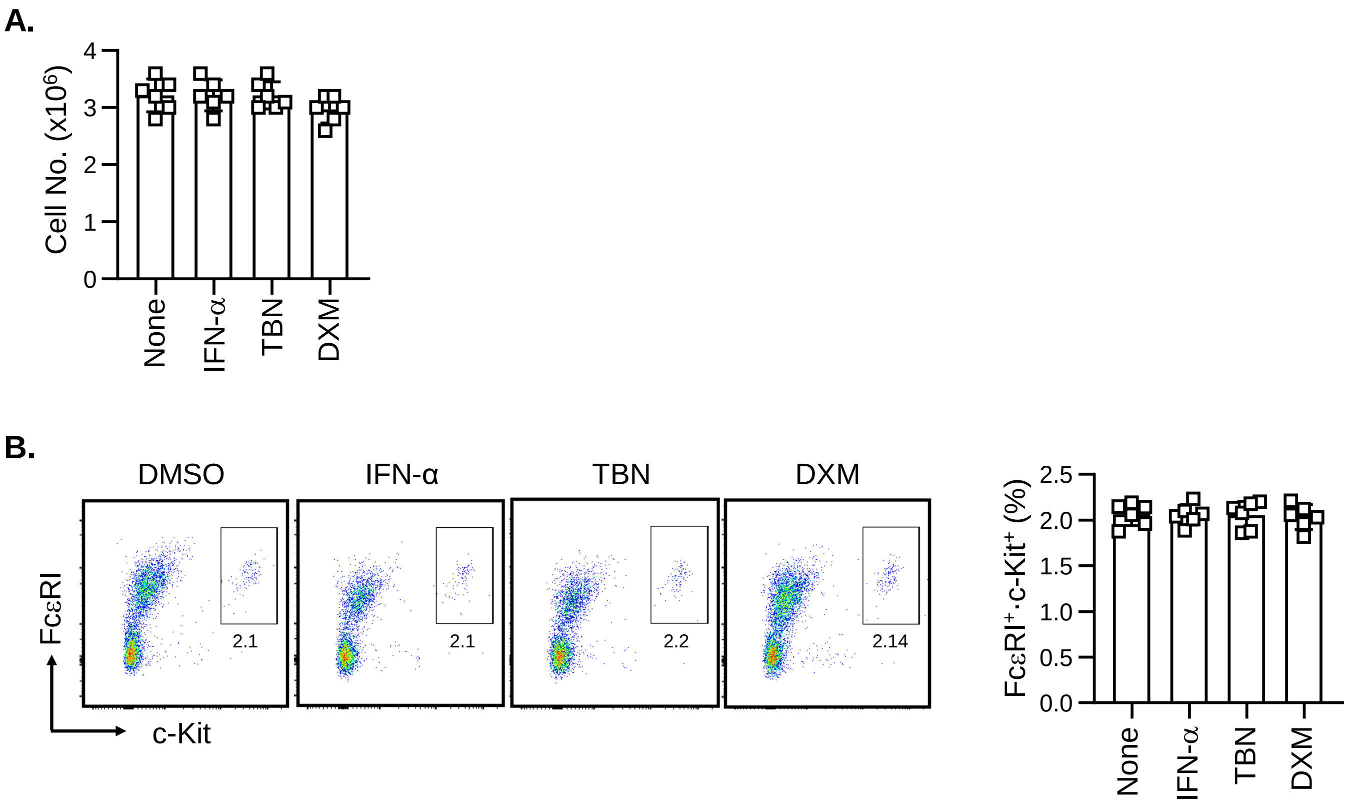

Figure S5. IFN-α, TBN, and DXM have no effect on total peritoneal cell counts or mast cell populations.

(A) No change in peritoneal cellularity after 2-day treatment (i.p.) with IFN-α (10 μg/kg), TBN (160 mg/kg), or DXM (2 mg/kg).

(B) No change in $Fc\varepsilon RI^{+}$·c-Kit^{+} peritoneal mast cell populations following IFN-α, TBN, or DXM treatment.

Figure S5
