# Supplementary material for: Impaired cortical actin dynamics via lanosterol-dependent HMG-CoA reductase downregulation mediates IFN-α-induced mast cell stabilization
Source: J Biol Chem. 2026 May 16;302(7):113161. doi: 10.1016/j.jbc.2026.113161 (PMC13279192; doi:10.1016/j.jbc.2026.113161)

All western blot Repeats

Note:The specific blots presented in the main figures are highlighted by red label.

1B IFN Concentration-HMGCR

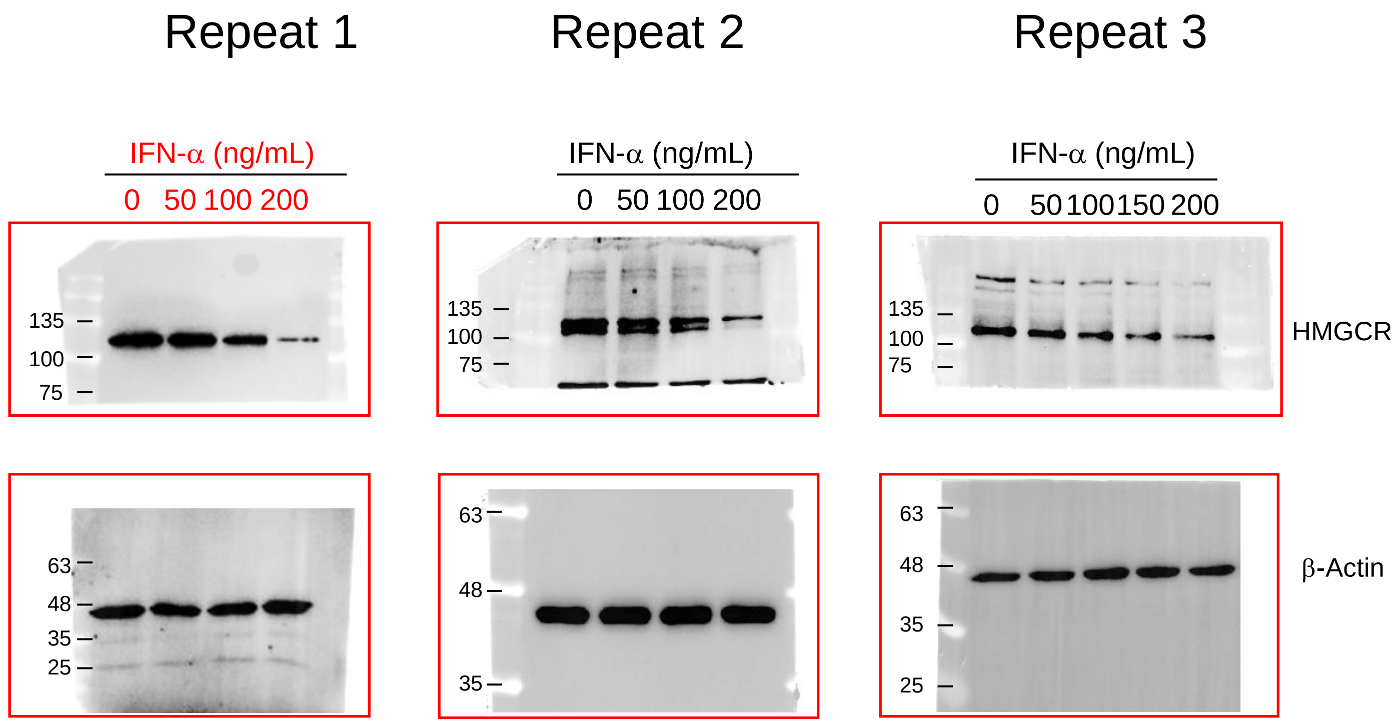

1B IFN Time -HMGCR

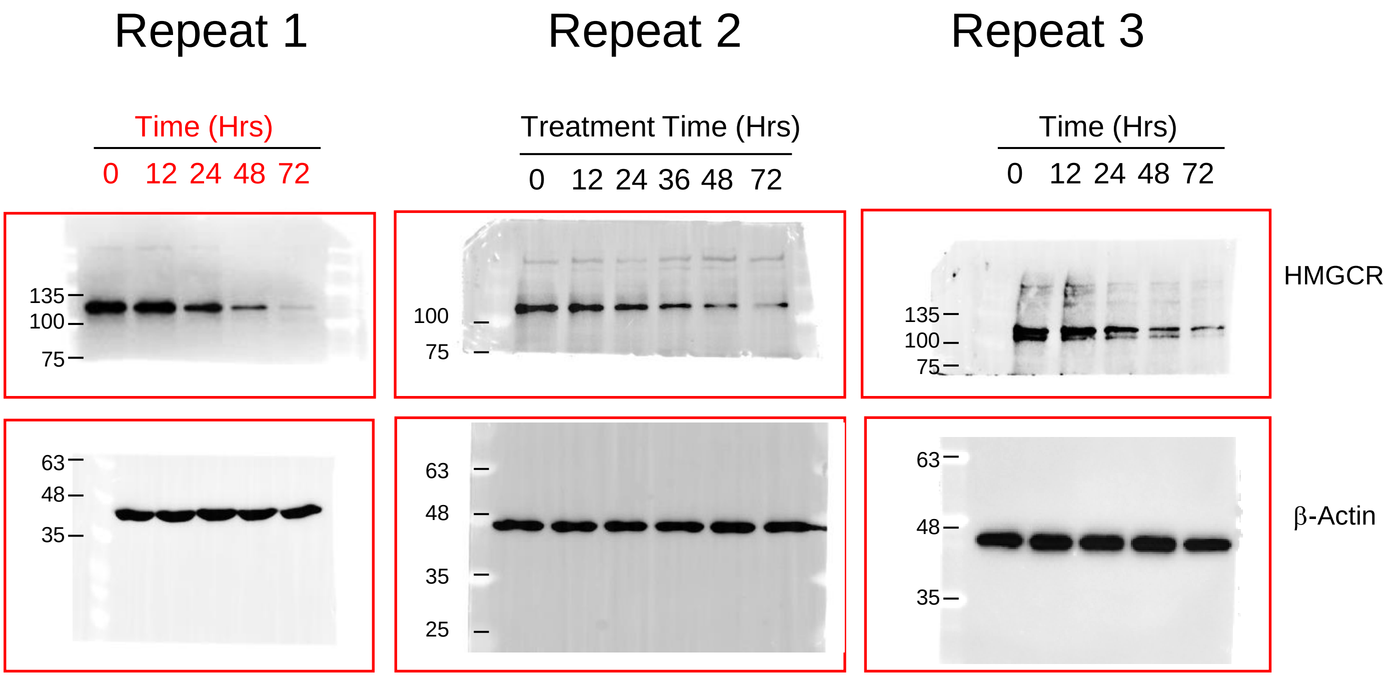

1C IFN Time -Ubiquitin

Repeat 1

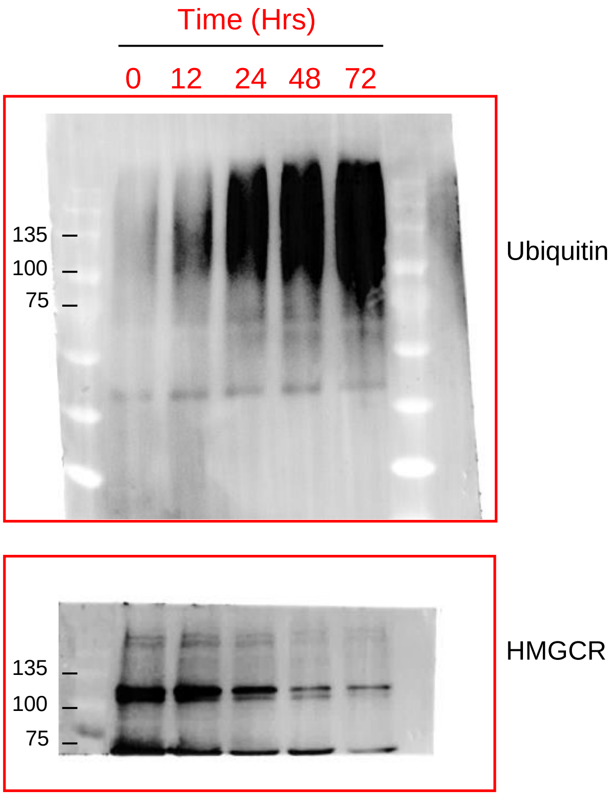

Repeat 2

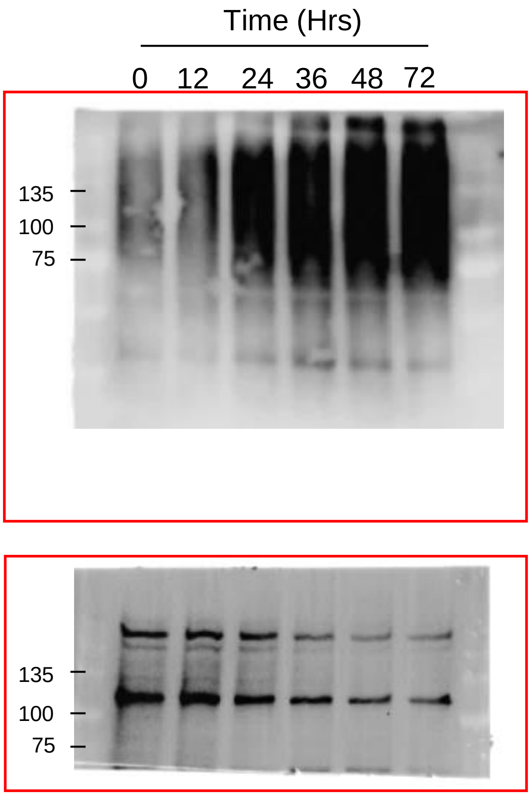

Repeat 3

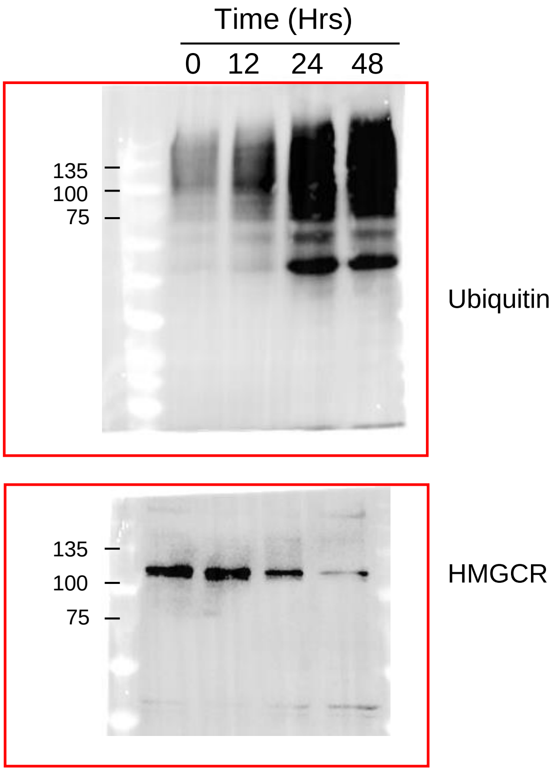

1E IFN+TBN HMGCR CYP51A1

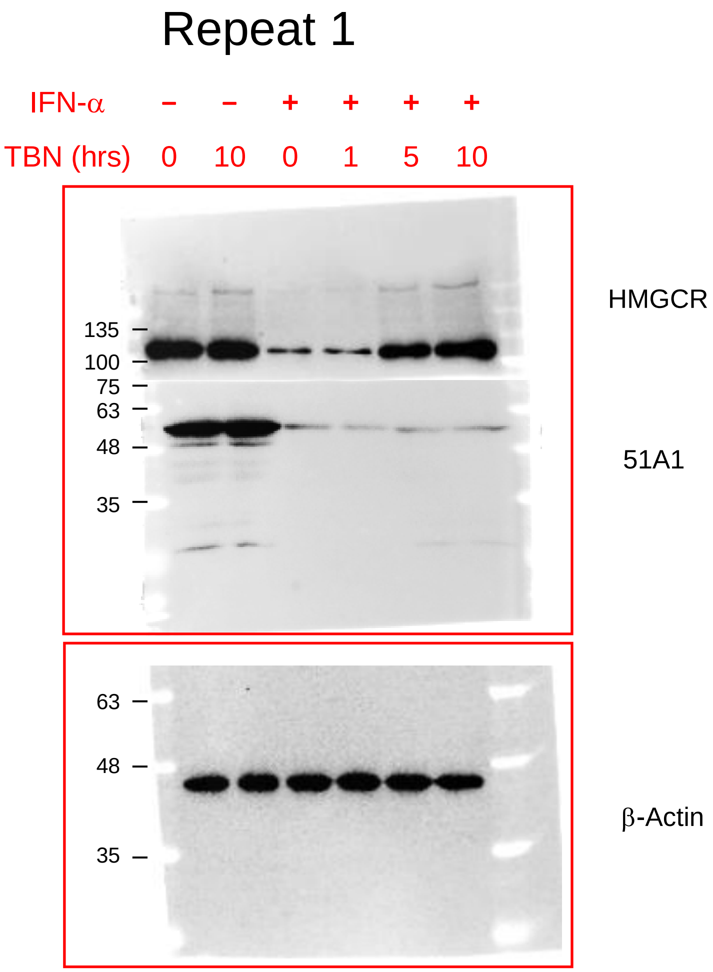

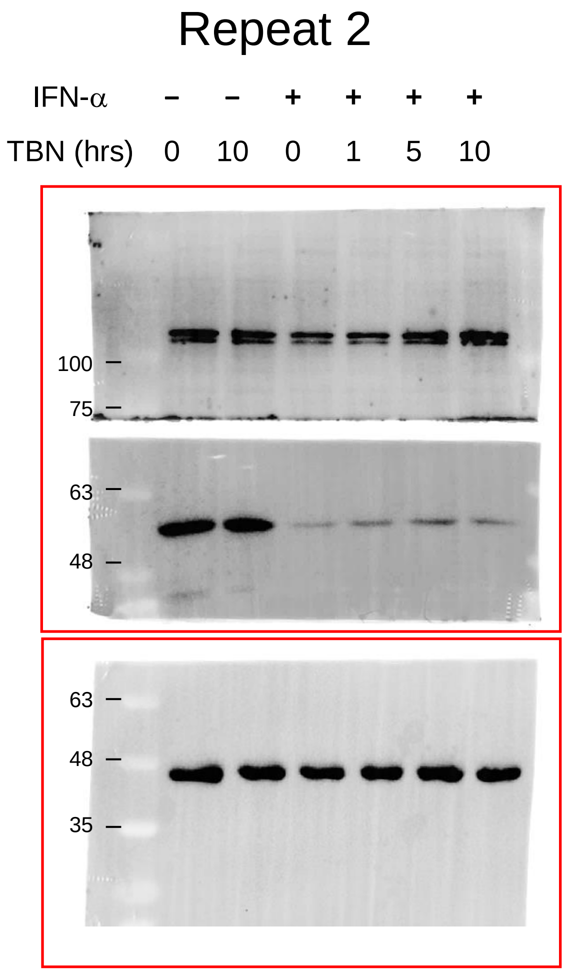

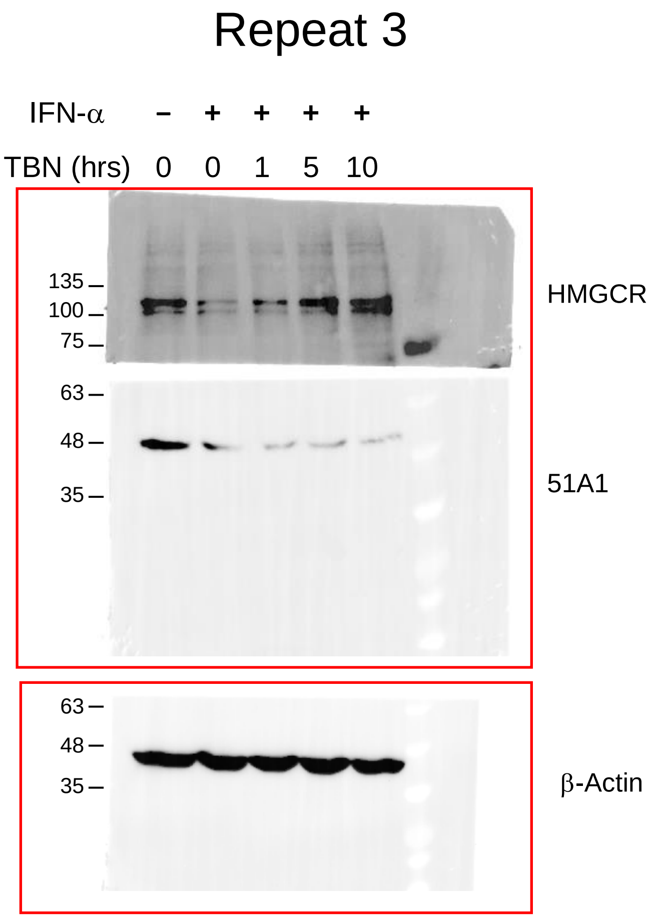

1E IFN+TBN Ubiquitin

Repeat 1 / Repeat 2

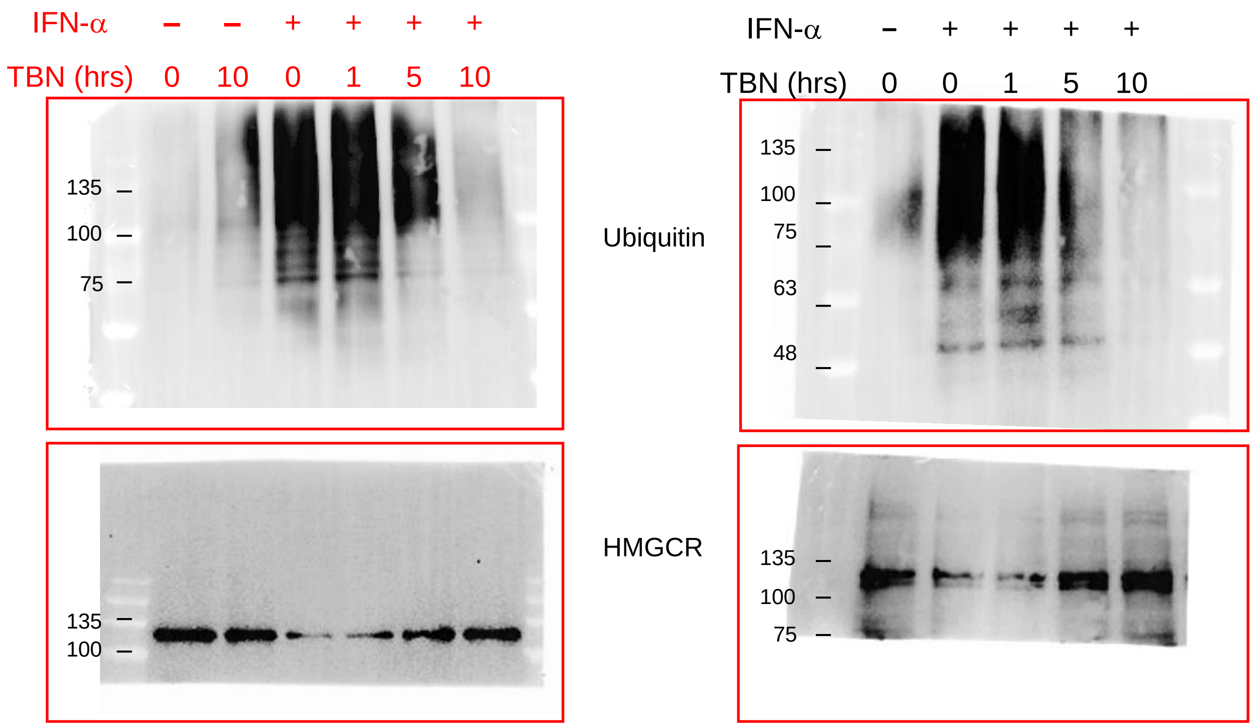

Repeat 3

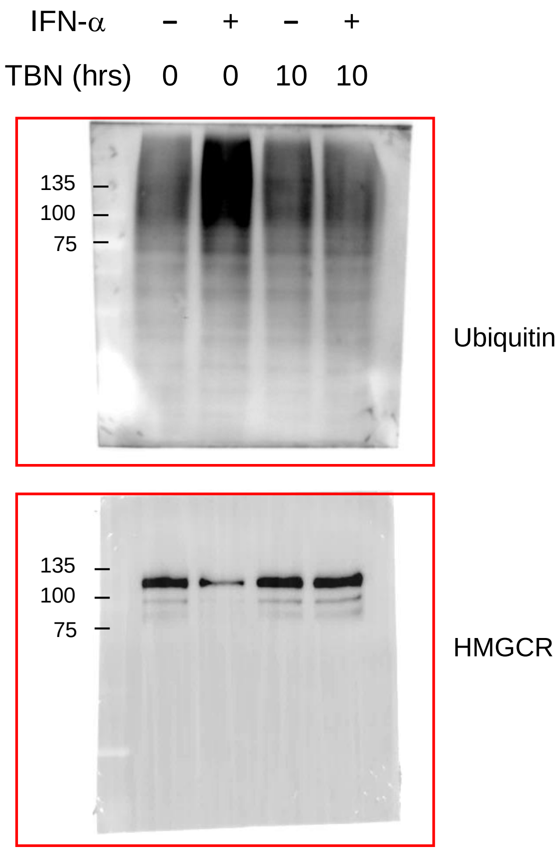

1G siRNA Time HMGCR CYP51A1

Repeat 1

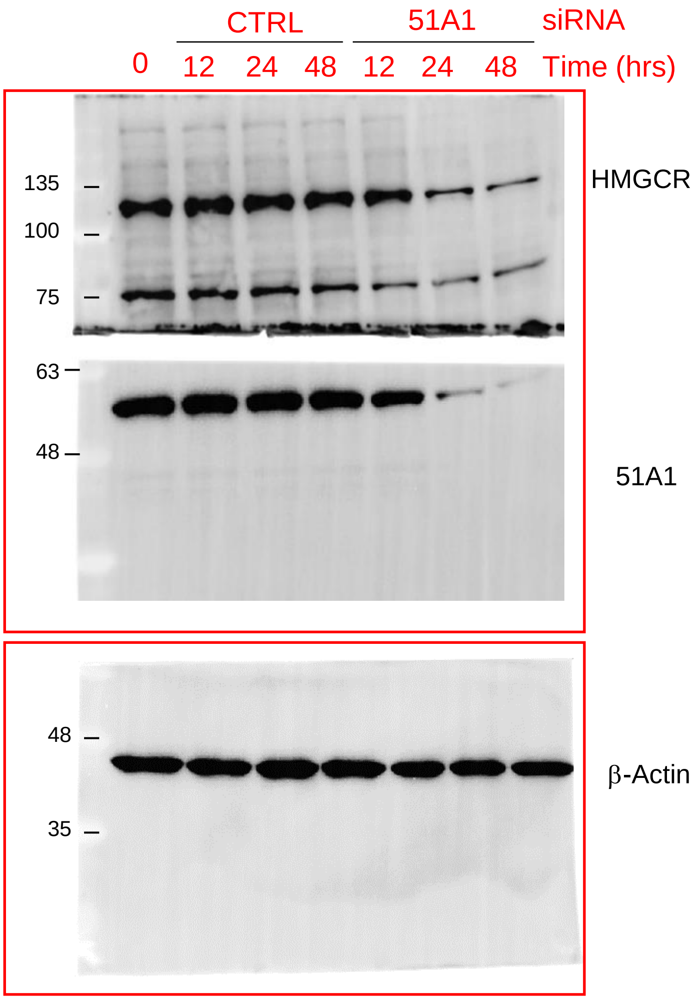

Repeat 2

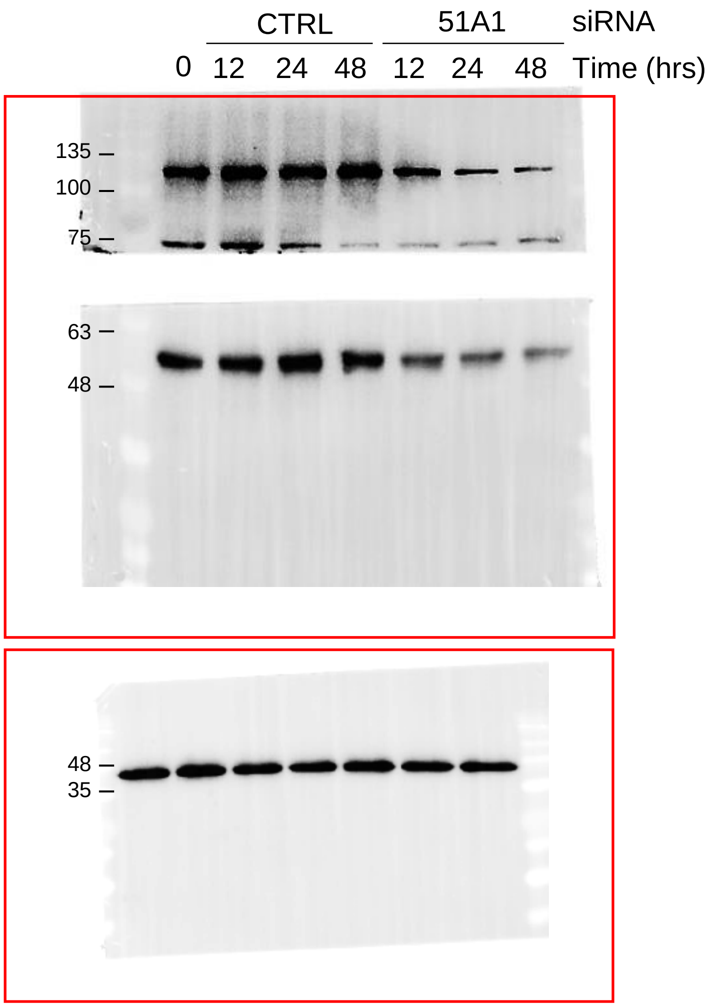

Repeat 3

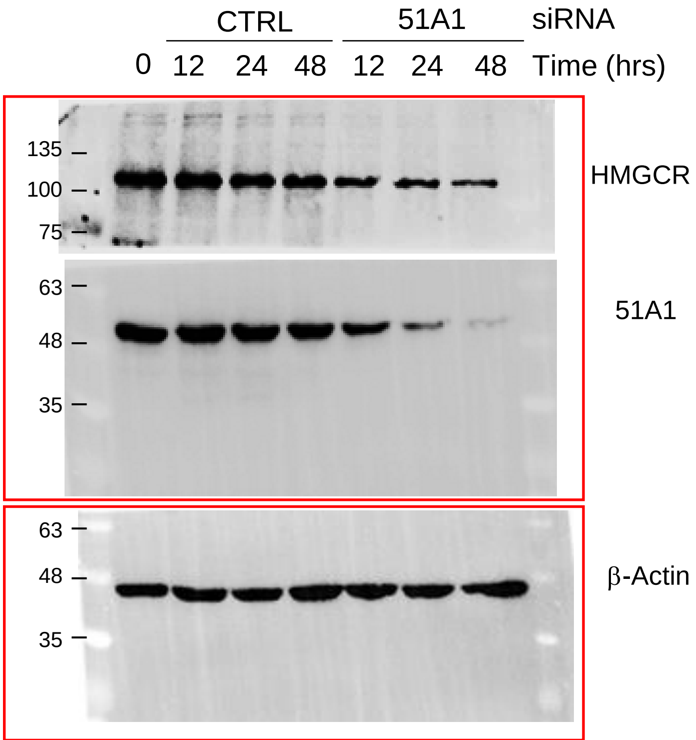

1G siRNA Time Ubiquitin

Repeat 1

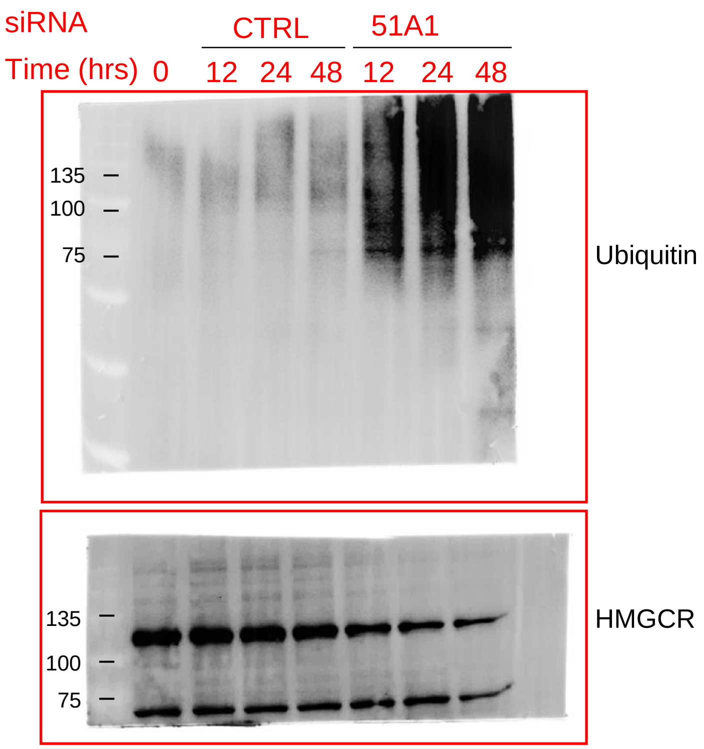

Repeat 2

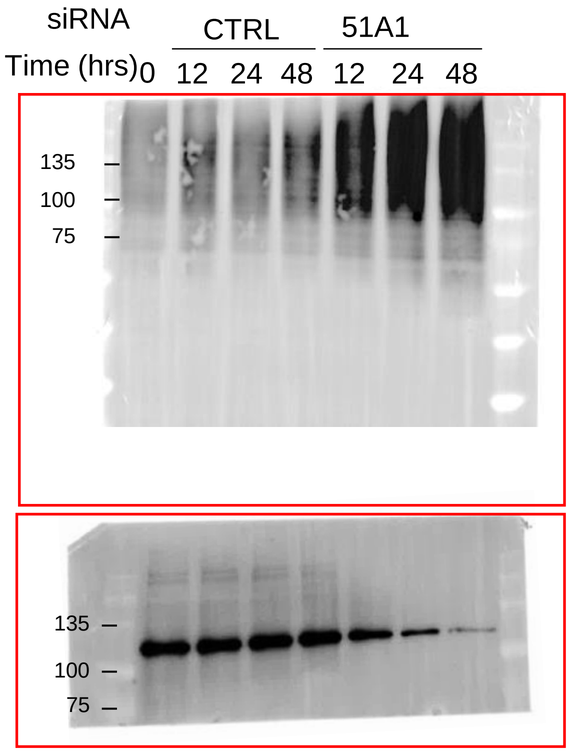

Repeat 3

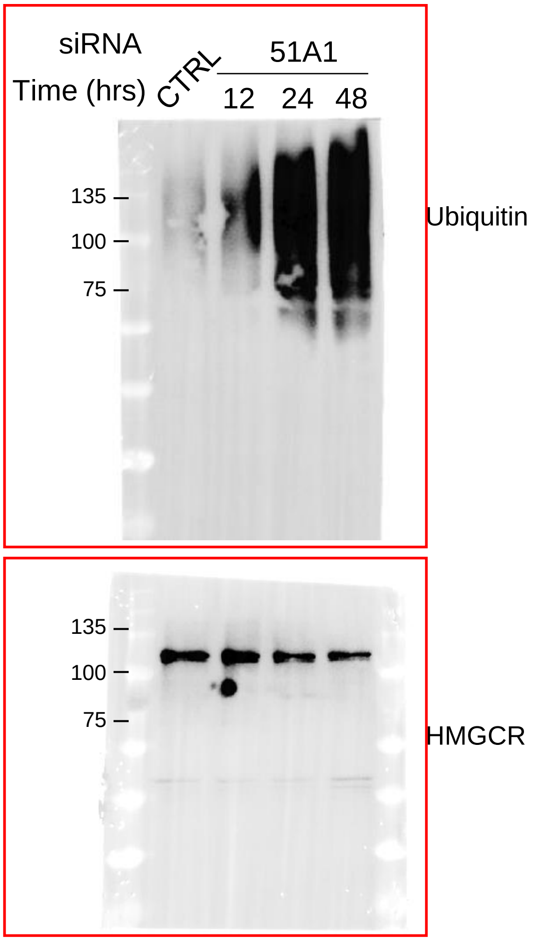

2H IFN

Repeat 1

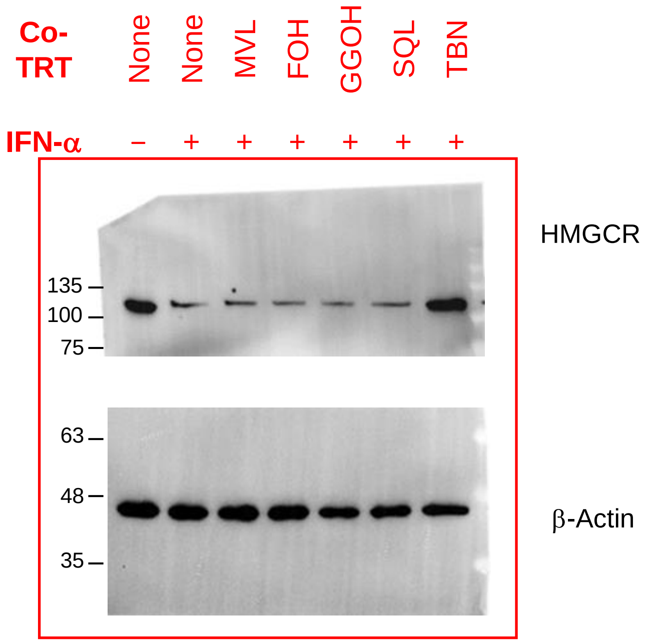

Repeat 2

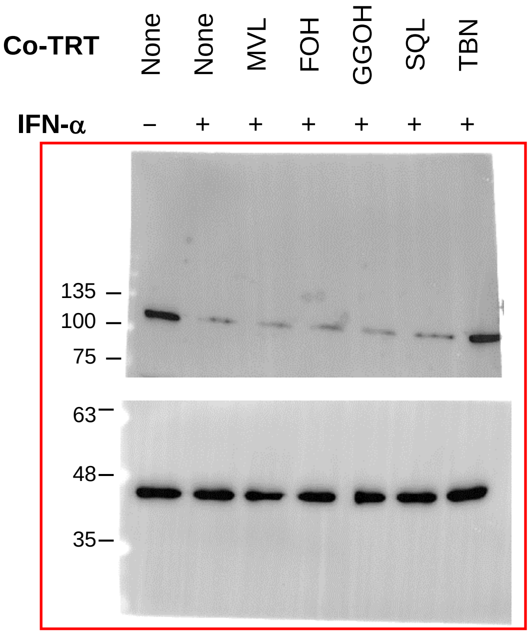

Repeat 3

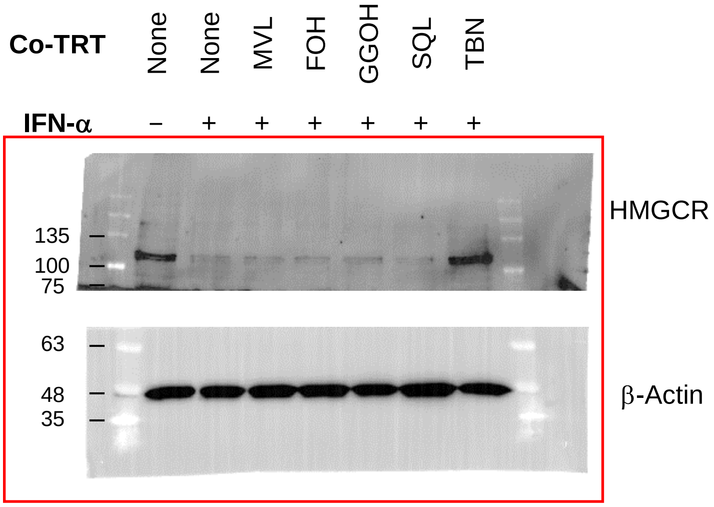

2H siRNA

Repeat 1

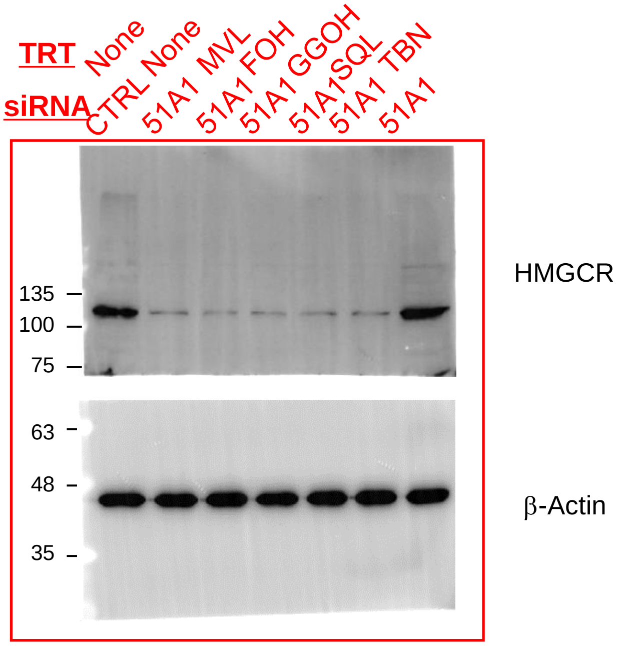

Repeat 2

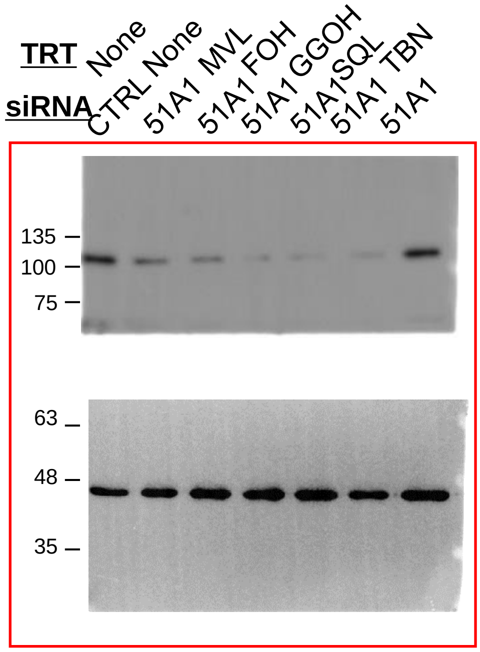

Repeat 3

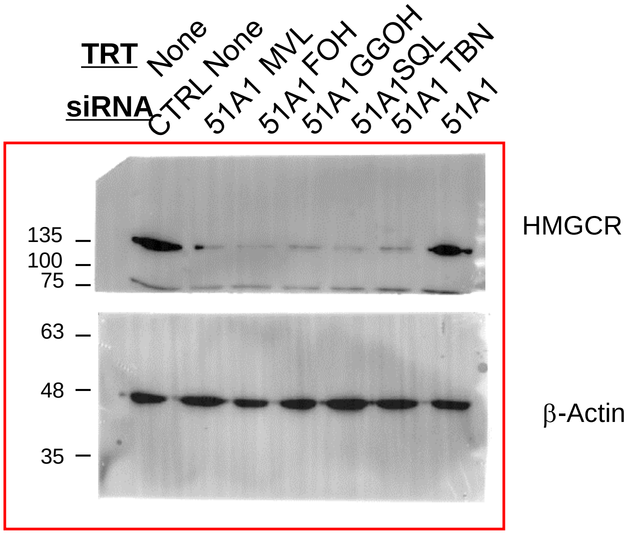

3E IFN LAT sigmalosome

Repeat 1

Co-TRT	None	None	None	None	MVL	FOH	GGOH	SQL	TBN
IFN-α	–	+	–	+	+	+	+	+	+
Ag	–	–	+	+	+	+	+	+	+

Repeat 2

Co-TRT	None	None	None	None	MVL	FOH	GGOH	SQL	TBN
IFN-α	–	+	–	+	+	+	+	+	+
Ag	–	–	+	+	+	+	+	+	+

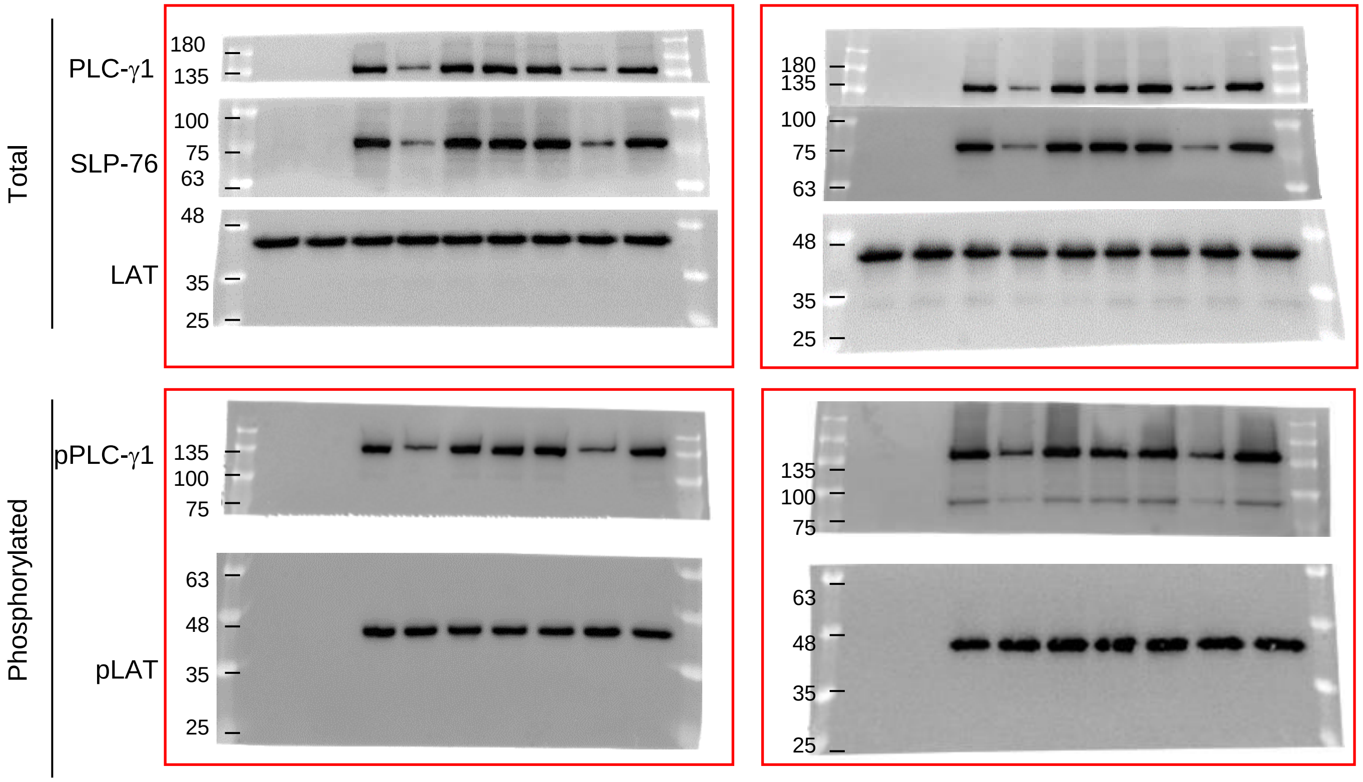

3E IFN LAT sigmalosome

Repeat 3

Co-TRT	None	None	None	None	MVL	FOH	GGOH	SQL	TBN
IFN-α	–	+	–	+	+	+	+	+	+
Ag	–	–	+	+	+	+	+	+	+

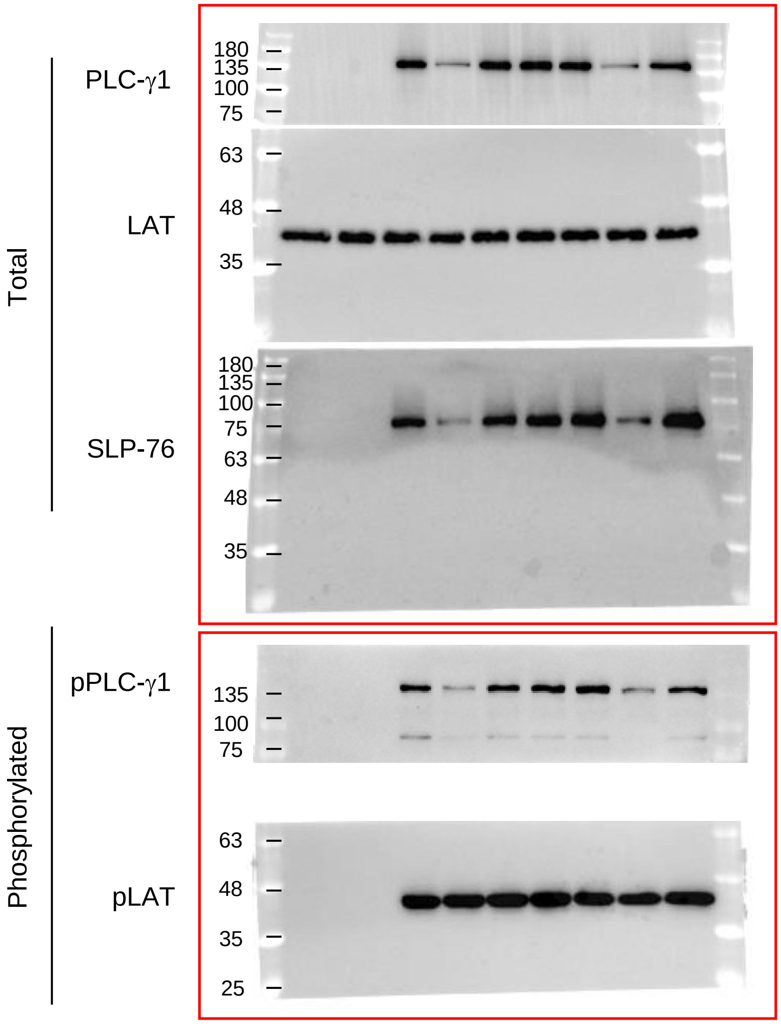

3F siRNA LAT sigmalosome

Repeat 1

Co-TRT	None	None	None	None	MVL	FOH	GGOH	SQL	TBN
SiRNA	–	+	–	+	+	+	+	+	+
Ag	–	–	+	+	+	+	+	+	+

Repeat 2

Co-TRT	None	None	None	None	MVL	FOH	GGOH	SQL	TBN
SiRNA	–	+	–	+	+	+	+	+	+
Ag	–	–	+	+	+	+	+	+	+

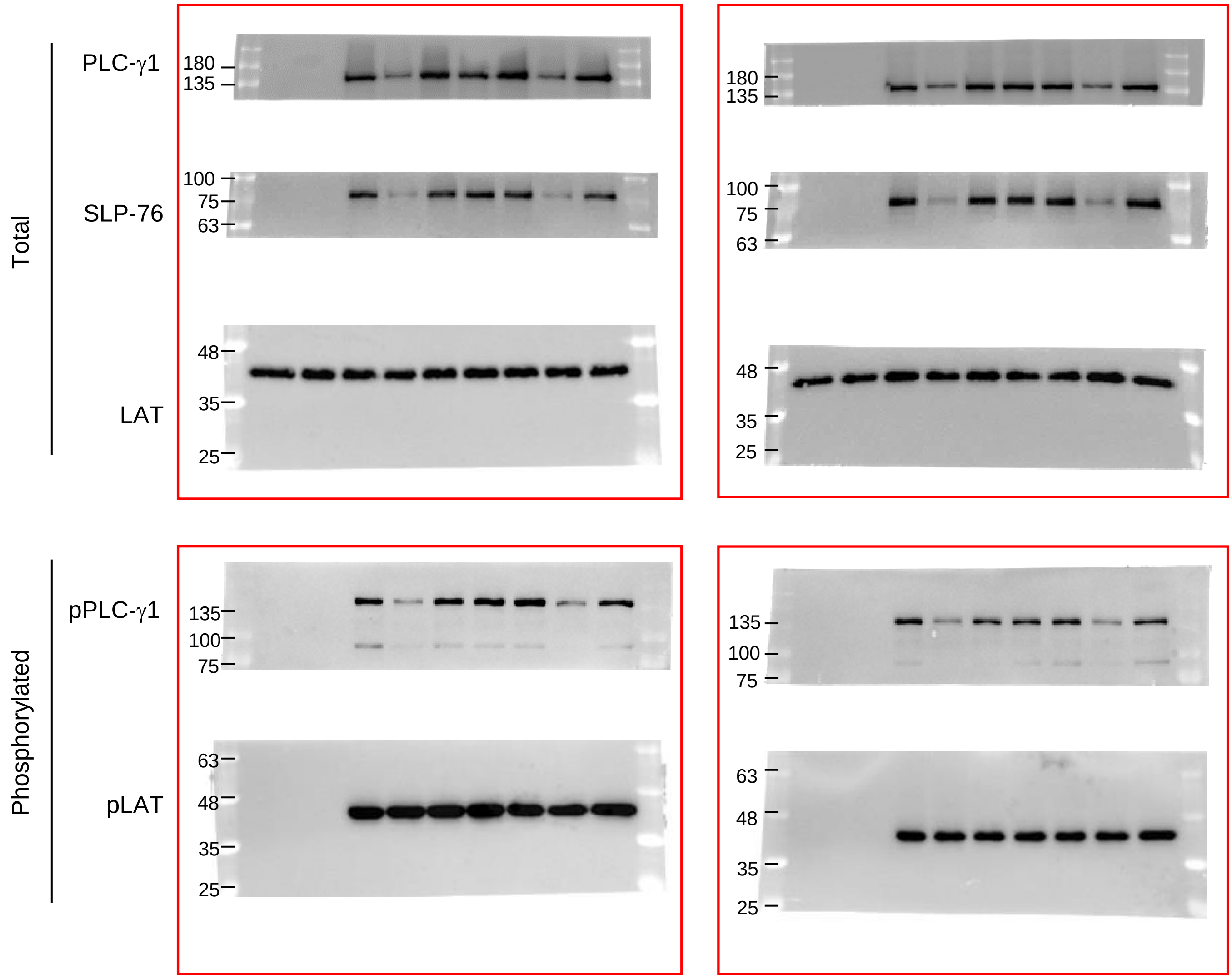

4C IFN Triton

Repeat 1

Co-TRT	None	None	None	None	MVL	FoH	GGoH	SQL	TBN
IFN-α	–	+	–	+	+	+	+	+	+
TX-100	–		+						

Repeat 2

Co-TRT	None	None	None	None	MVL	FoH	GGoH	SQL	TBN
IFN-α	–	+	–	+	+	+	+	+	+
TX-100	–		+						

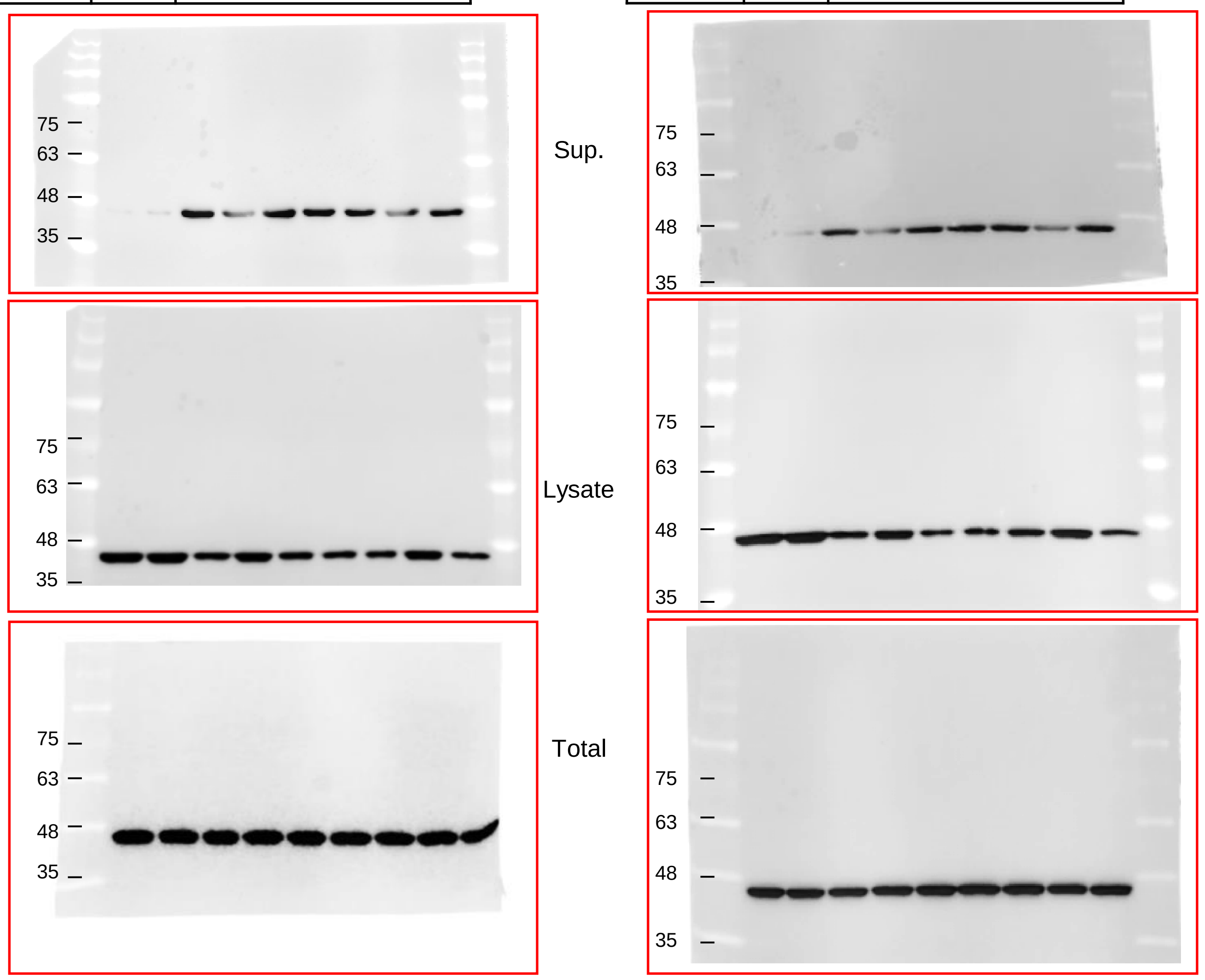

4C IFN Triton

Repeat 3

Co-TRT	None	None	None	None	MVL	FoH	GGoH	SQL	TBN
IFN-α	–	+	–	+	+	+	+	+	+
TX-100	–		+						

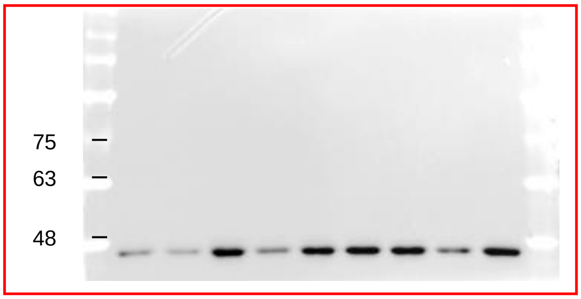

Sup.

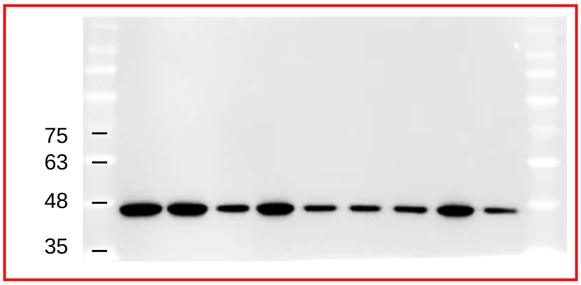

Pellet

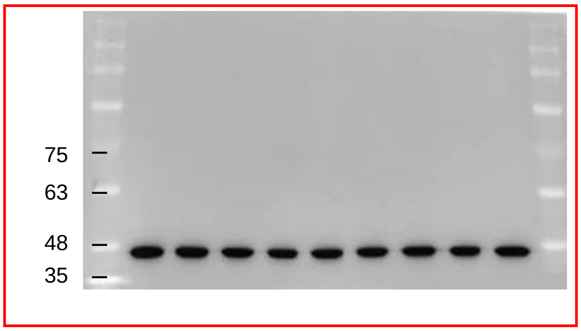

Total

5B IFN+CD Triton

Repeat 1

CD (nM)	0	20	0	3	20
IFN-α	–	–	+	+	+
TX-100	+	+	+	+	+

Repeat 2

CD (nM)	0	0	3	20	0	0	3	3	20	20
IFN-α	–	+			–	+	–	+	–	+
TX-100	–				+					

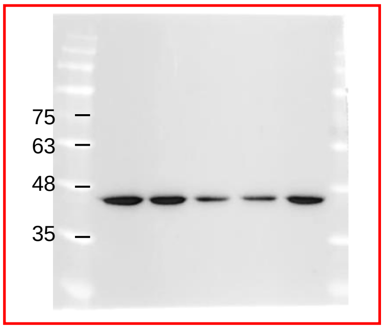

Sup.

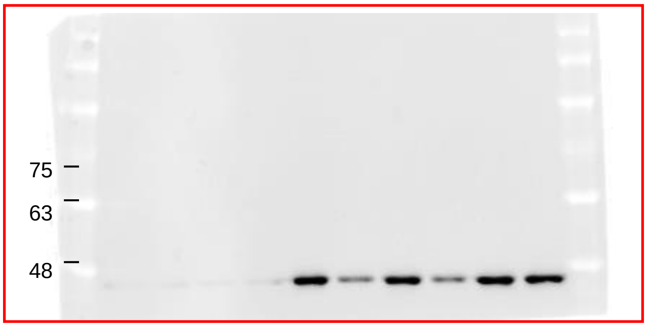

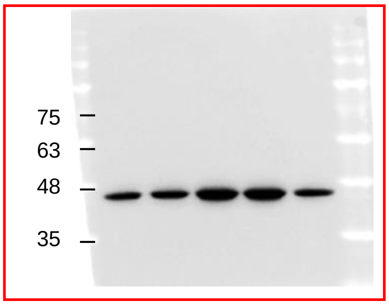

Lysate

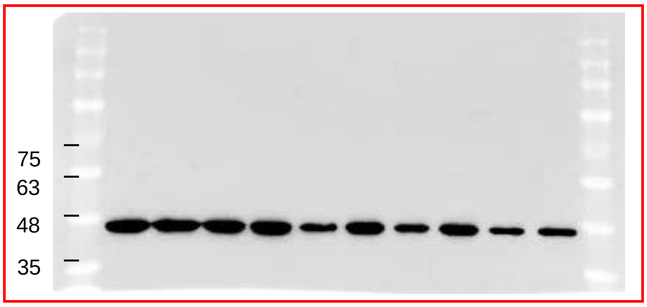

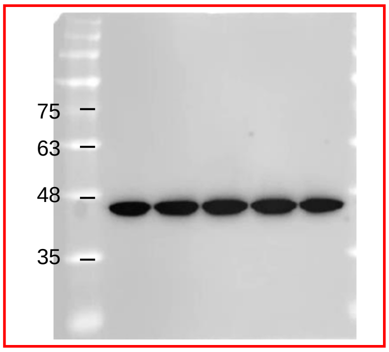

Total

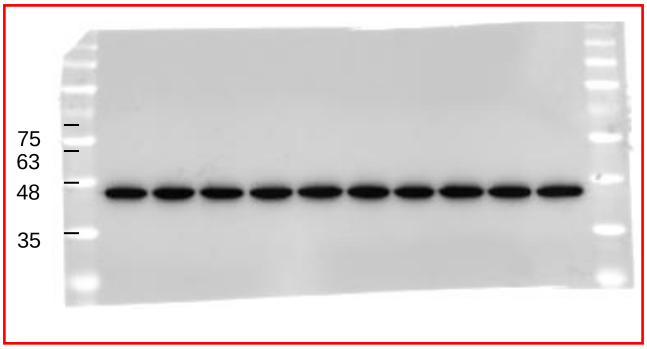

5B IFN+CD Triton

Repeat 3

CD (nM)	0	0	3	20	0	0	3	3	20	20
IFN-α	–	+			–	+	–	+	–	+
TX-100	–				+					

Repeat 4

CD (nM)	0	0	3	20	0	0	3	3	20	20
IFN-α	–	+			–	+	–	+	–	+
TX-100	–				+					

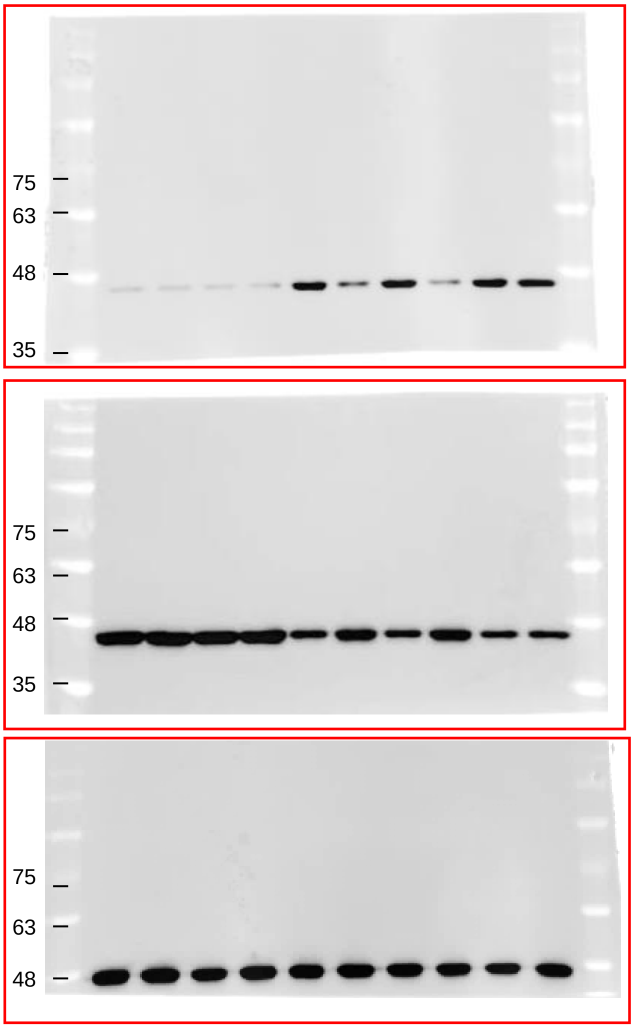

Sup.

Lysate

Total

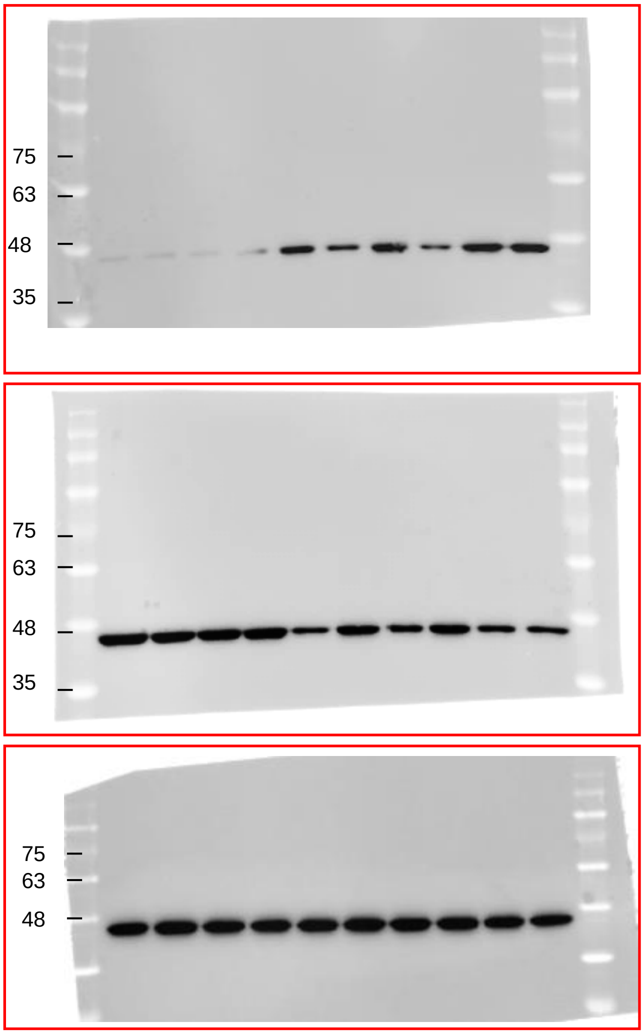

5E IFN+CD LAT Signalosome

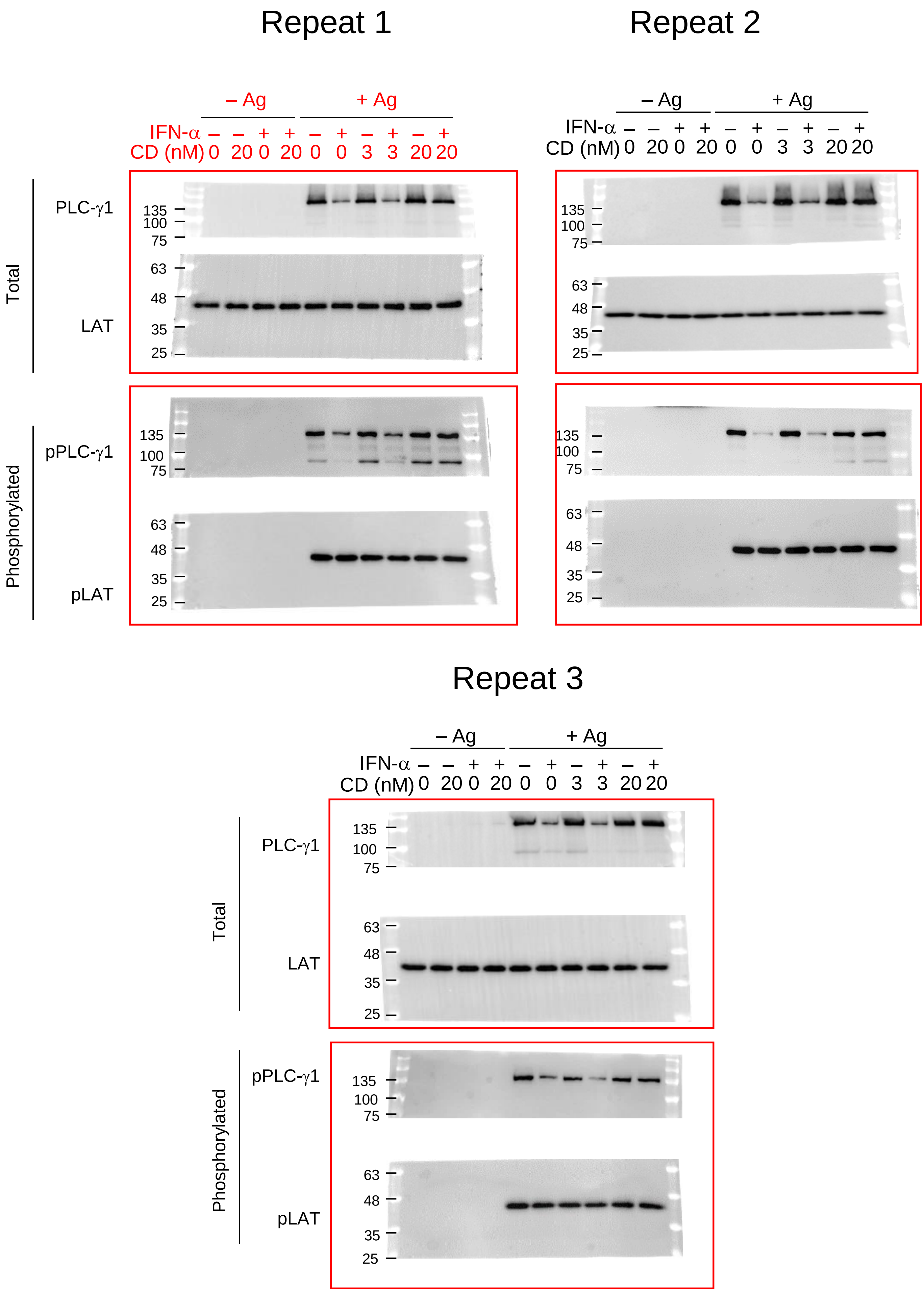

S1A IFN concentration CY51A1

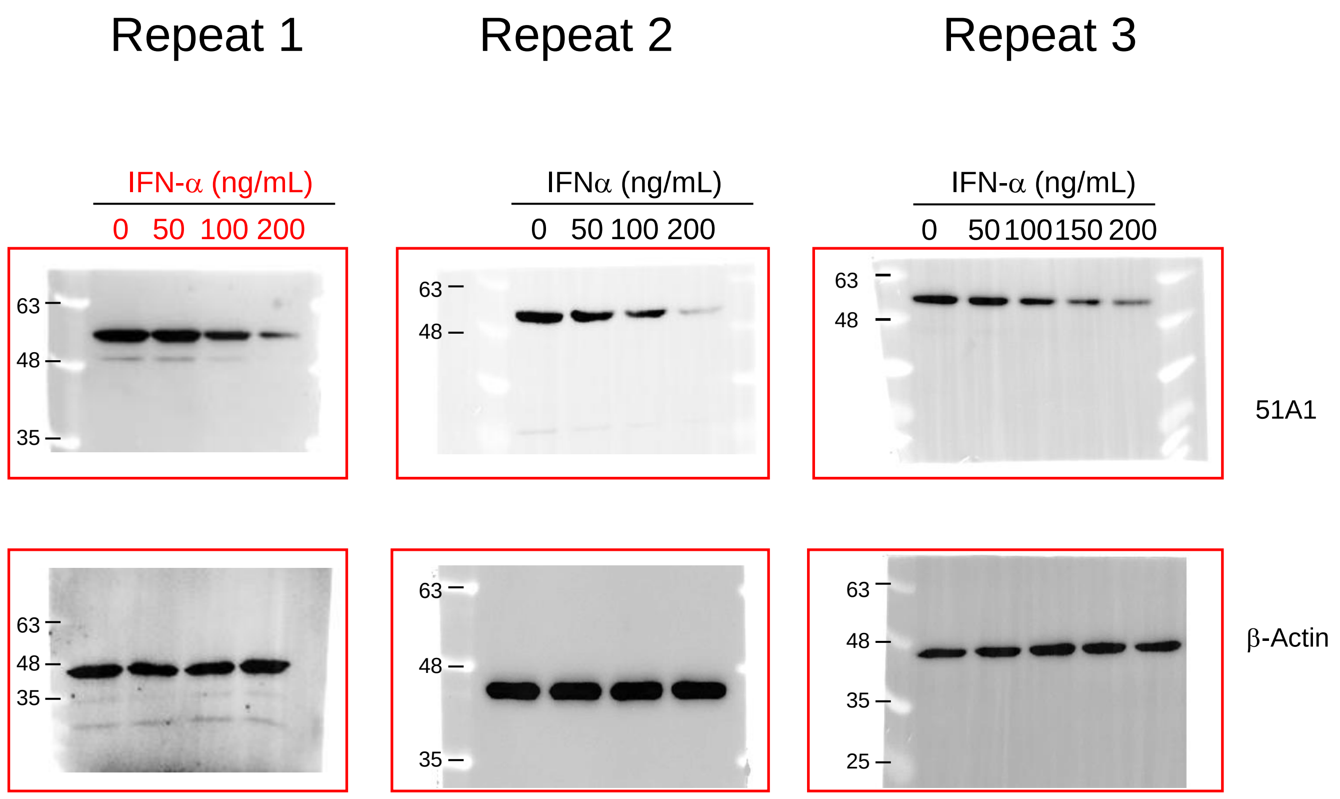

S1A IFN Time CY51A1

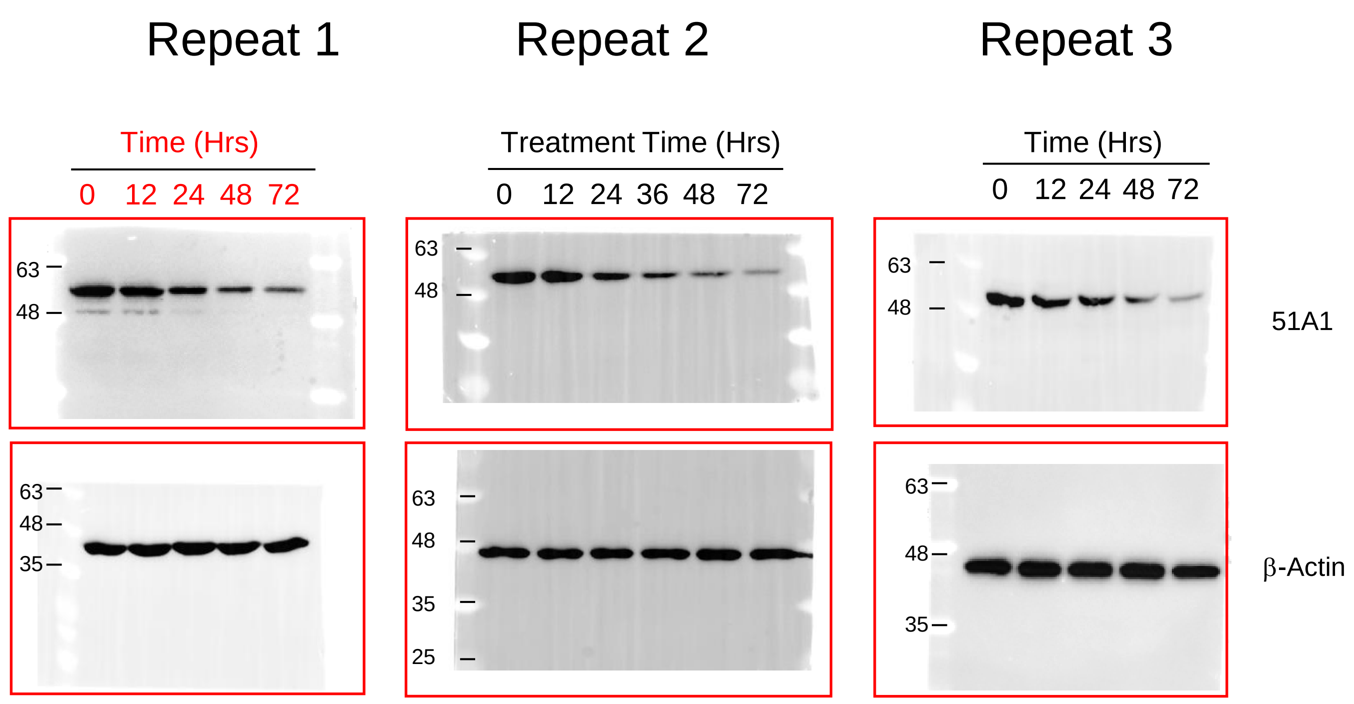

S2A IFN Signaling ca free

Repeat 1

Co-TRT	None	None	None	None	MVL	FOH	GGOH	SQL	TBN
IFN-α	–	+	–	+	+	+	+	+	+
Ag	–	–	+	+	+	+	+	+	+

Repeat 2

Co-TRT	None	None	None	None	MVL	FOH	GGOH	SQL	TBN
IFN-α	–	+	–	+	+	+	+	+	+
Ag	–	–	+	+	+	+	+	+	+

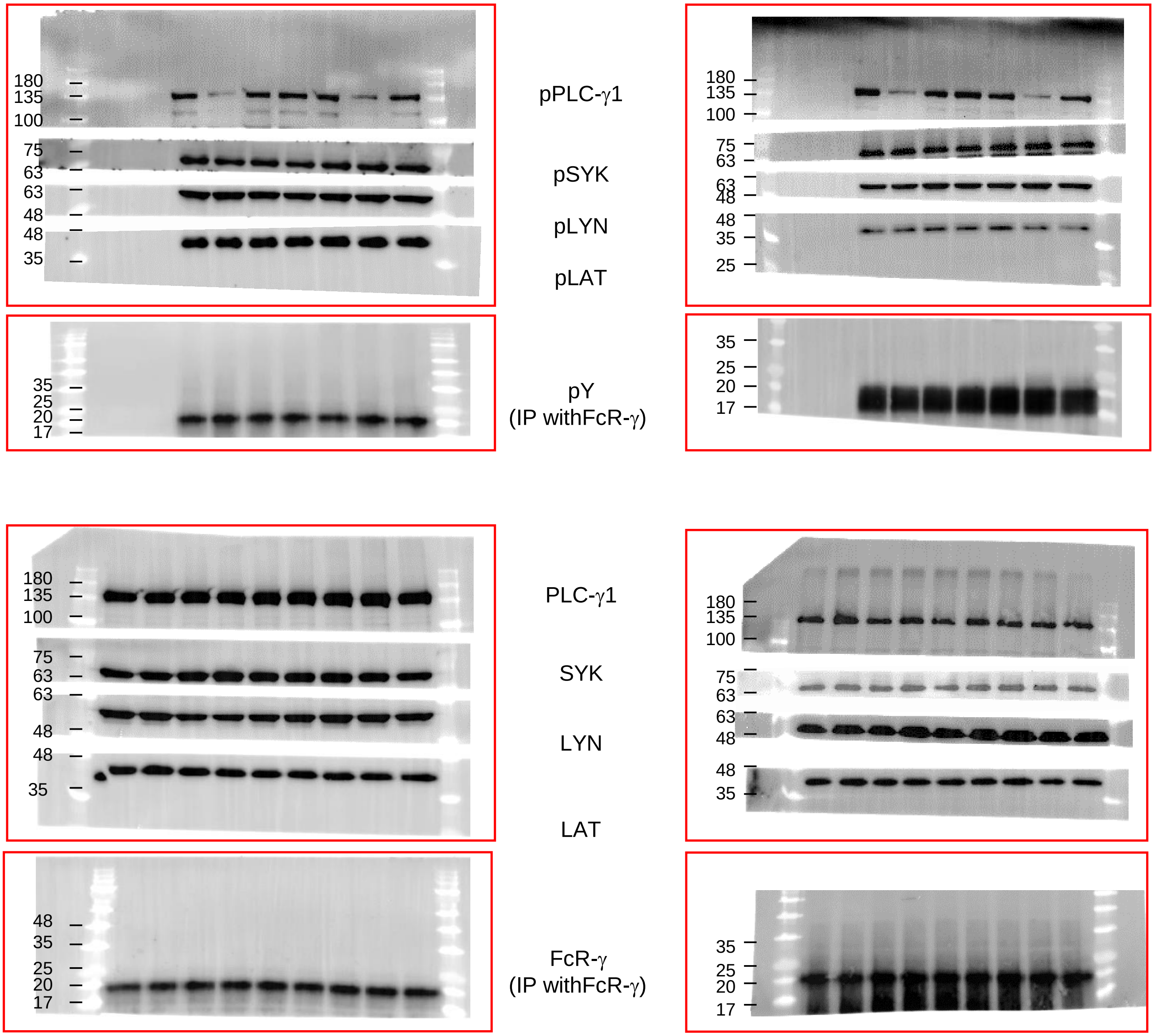

S2A IFN Signaling ca free

Repeat 3

Co-TRT	None	None	None	None	MVL	FOH	GGOH	SQL	TBN
IFN-α	–	+	–	+	+	+	+	+	+
Ag	–	–	+	+	+	+	+	+	+

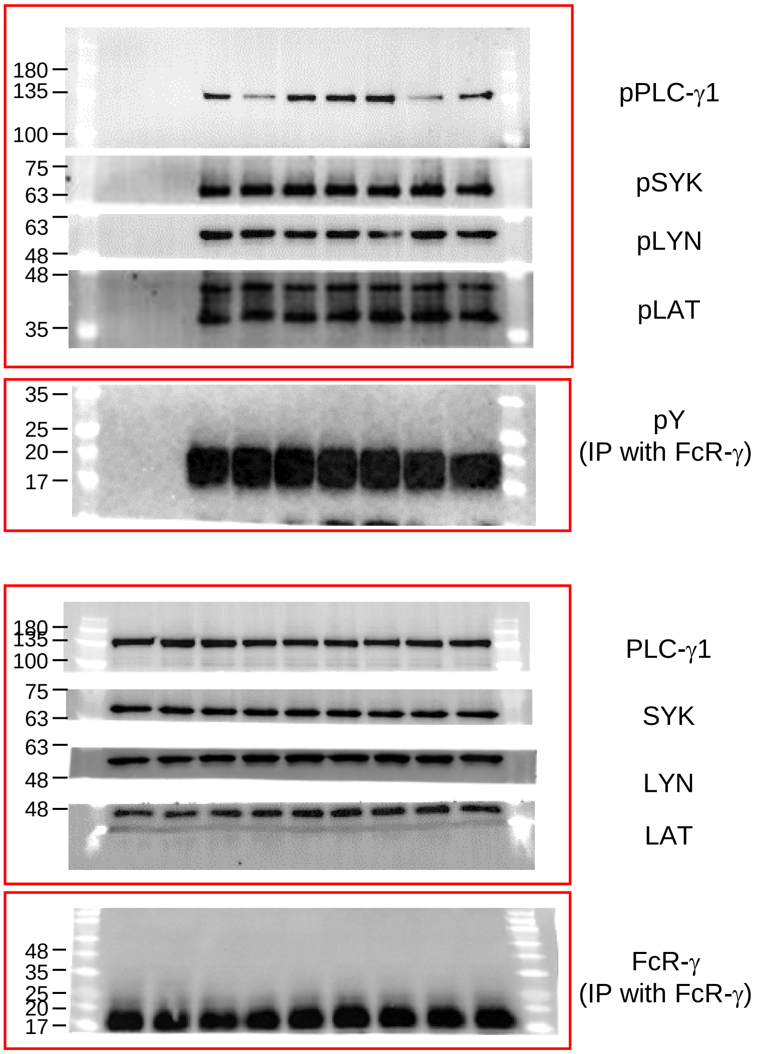

S2B siRNA Signaling ca free

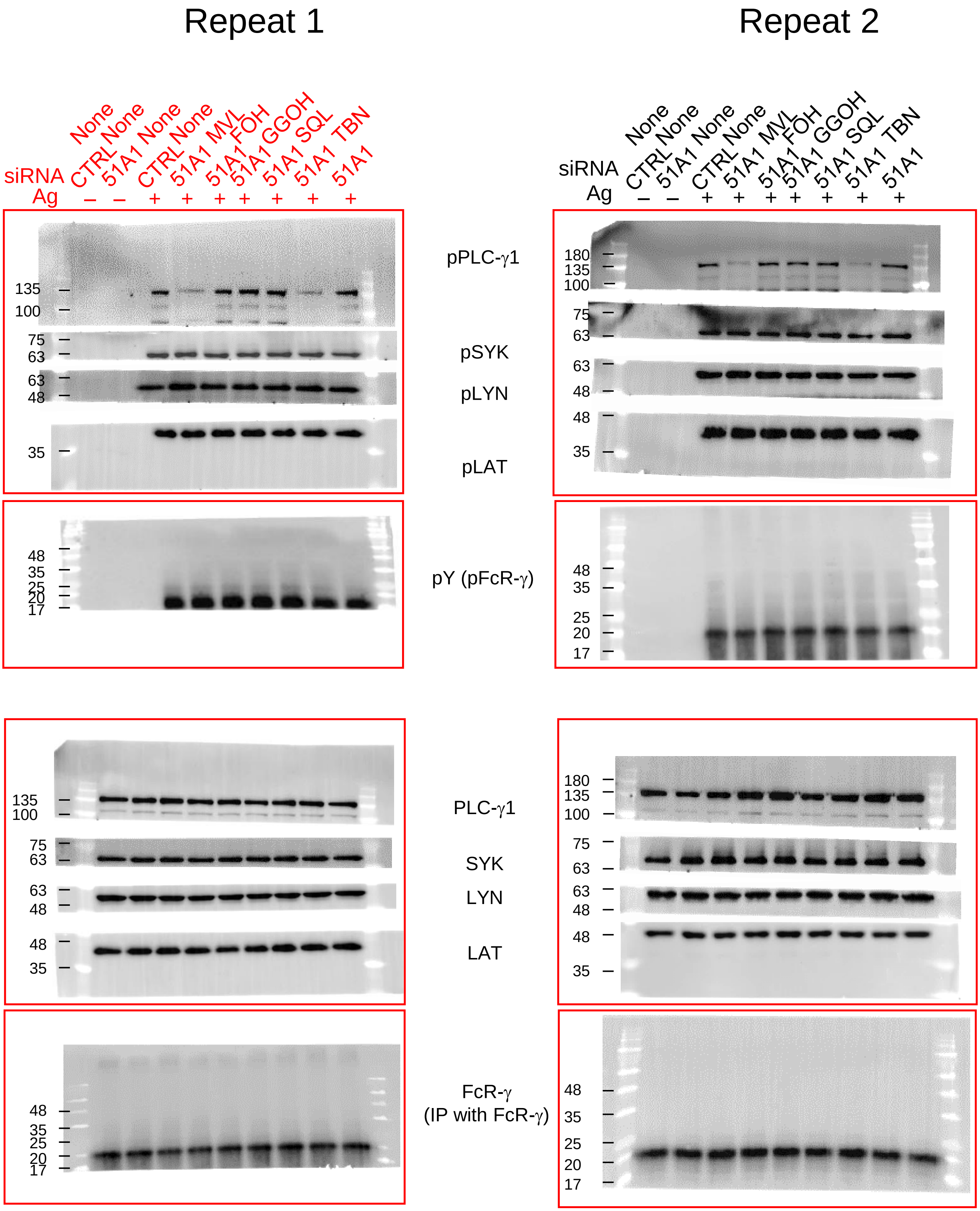

S3B CYP51A1 Triton

Repeat 1

Co-TRT	None	None	None	None	MVL	FoH	GGoH	SQL	TBN
51A1	–	+	–	+	+	+	+	+	+
TX-100	–		+						

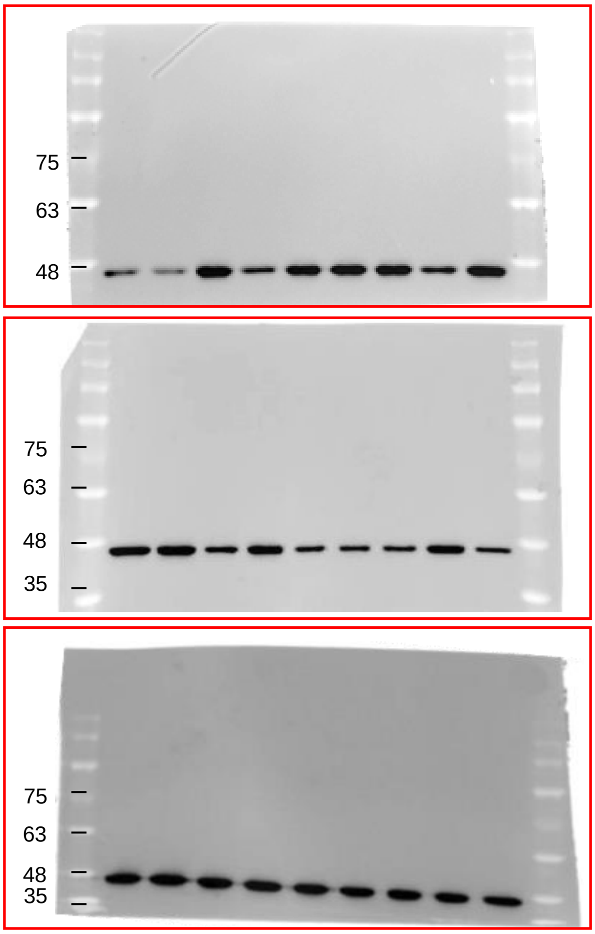

Sup.

Lysate

Total

Repeat 2

Co-TRT	None	None	None	None	MVL	FoH	GGoH	SQL	TBN
51A1	–	+	–	+	+	+	+	+	+
TX-100	–		+						

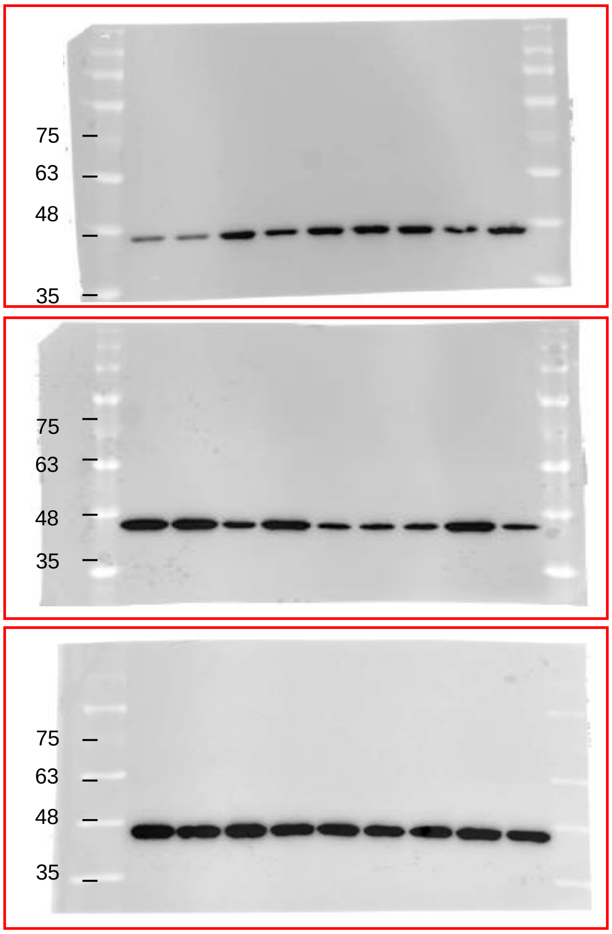

S3B CYP51A1 Triton

Repeat 3

Co-TRT	None	None	None	None	MVL	FoH	GGoH	SQL	TBN
51A1	–	+	–	+	+	+	+	+	+
TX-100	–		+						

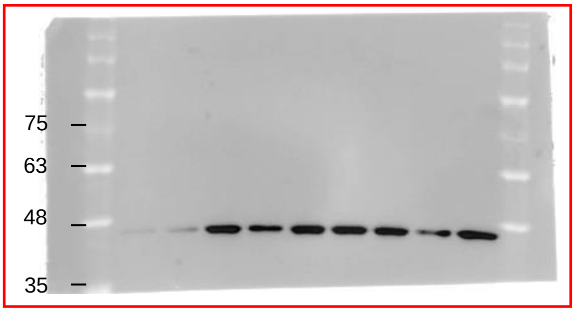

Sup.

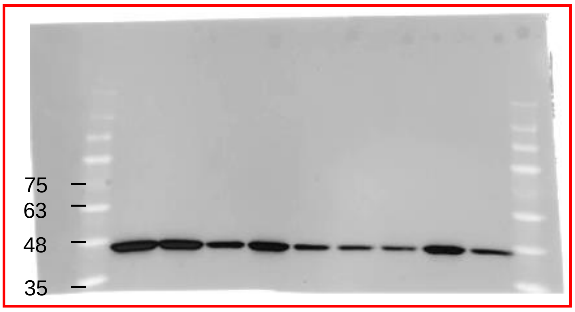

Lysate

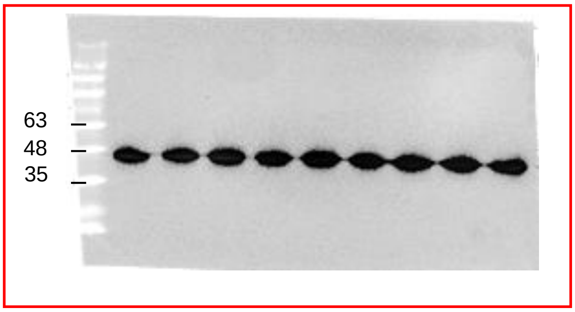

Total

S4A siRNA+CD Triton

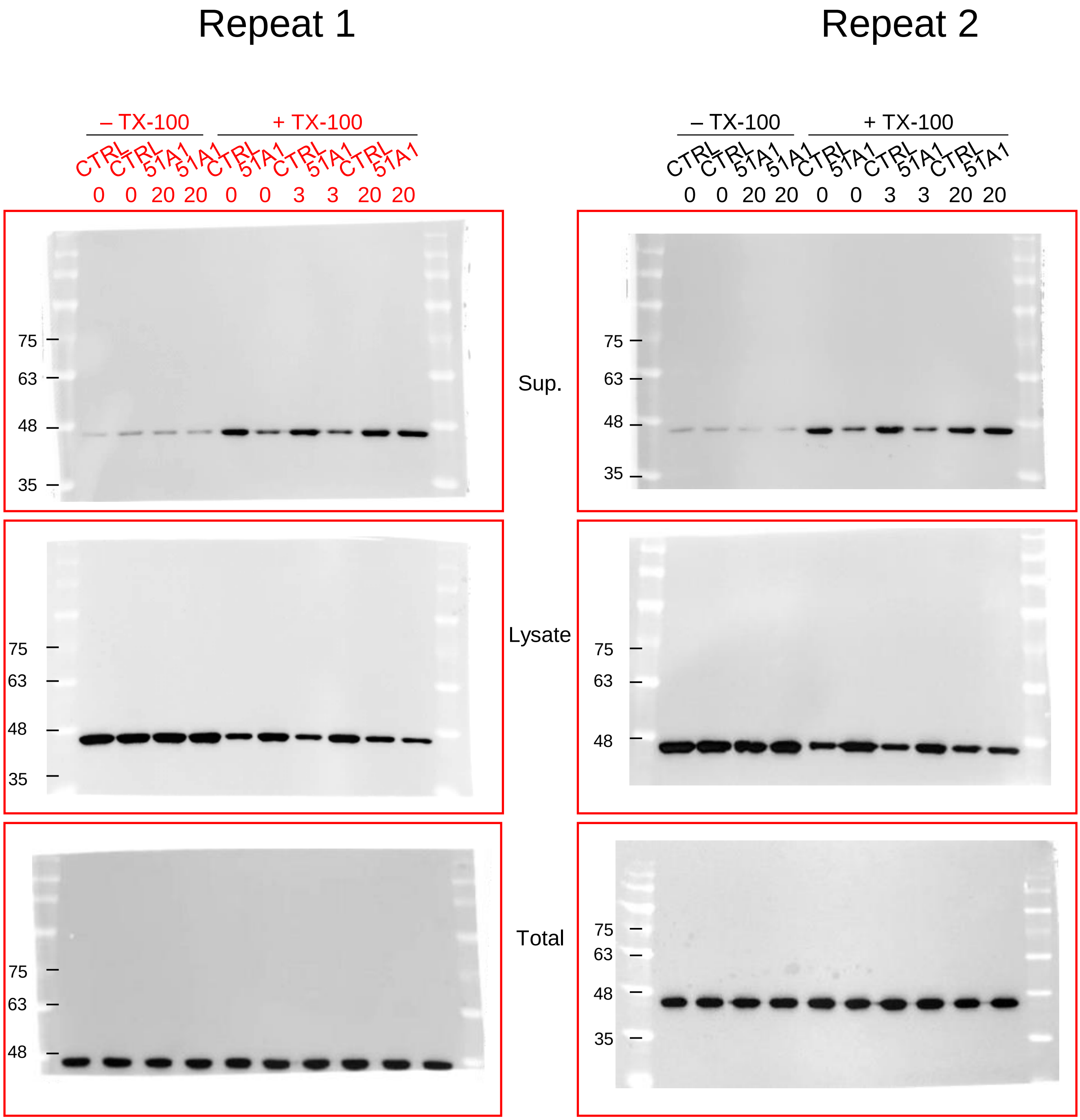

S4A siRNA+CD Triton

Repeat 3

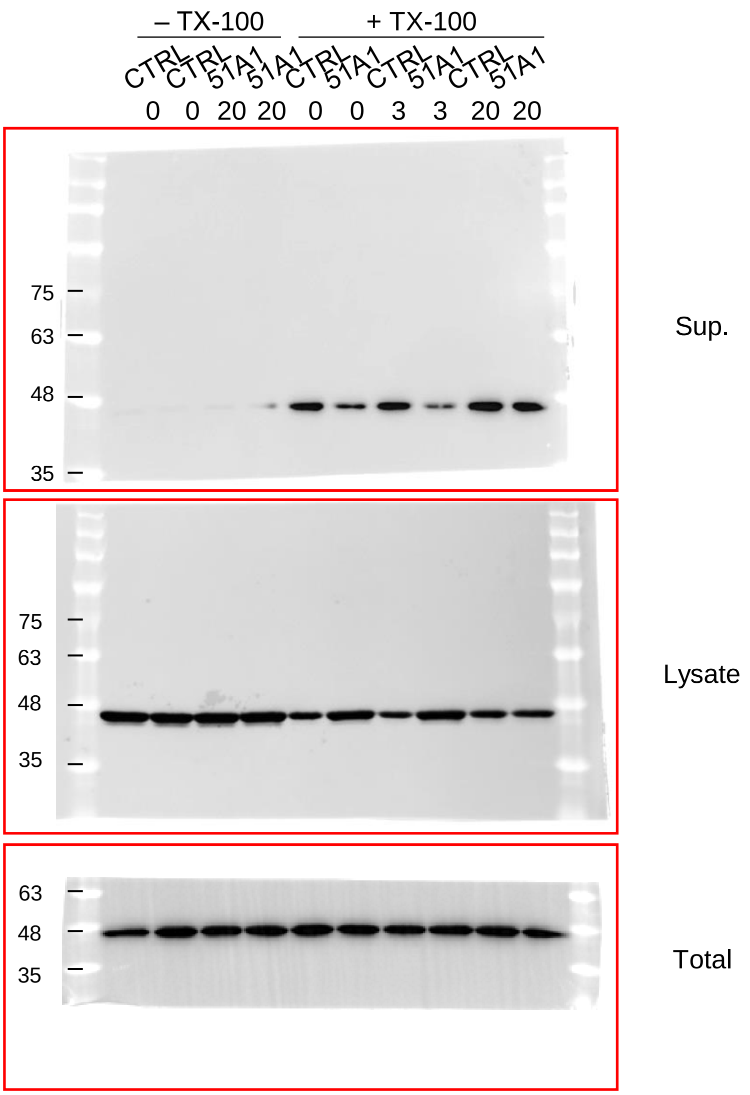

S4D siRNA+CD LAT signalosome

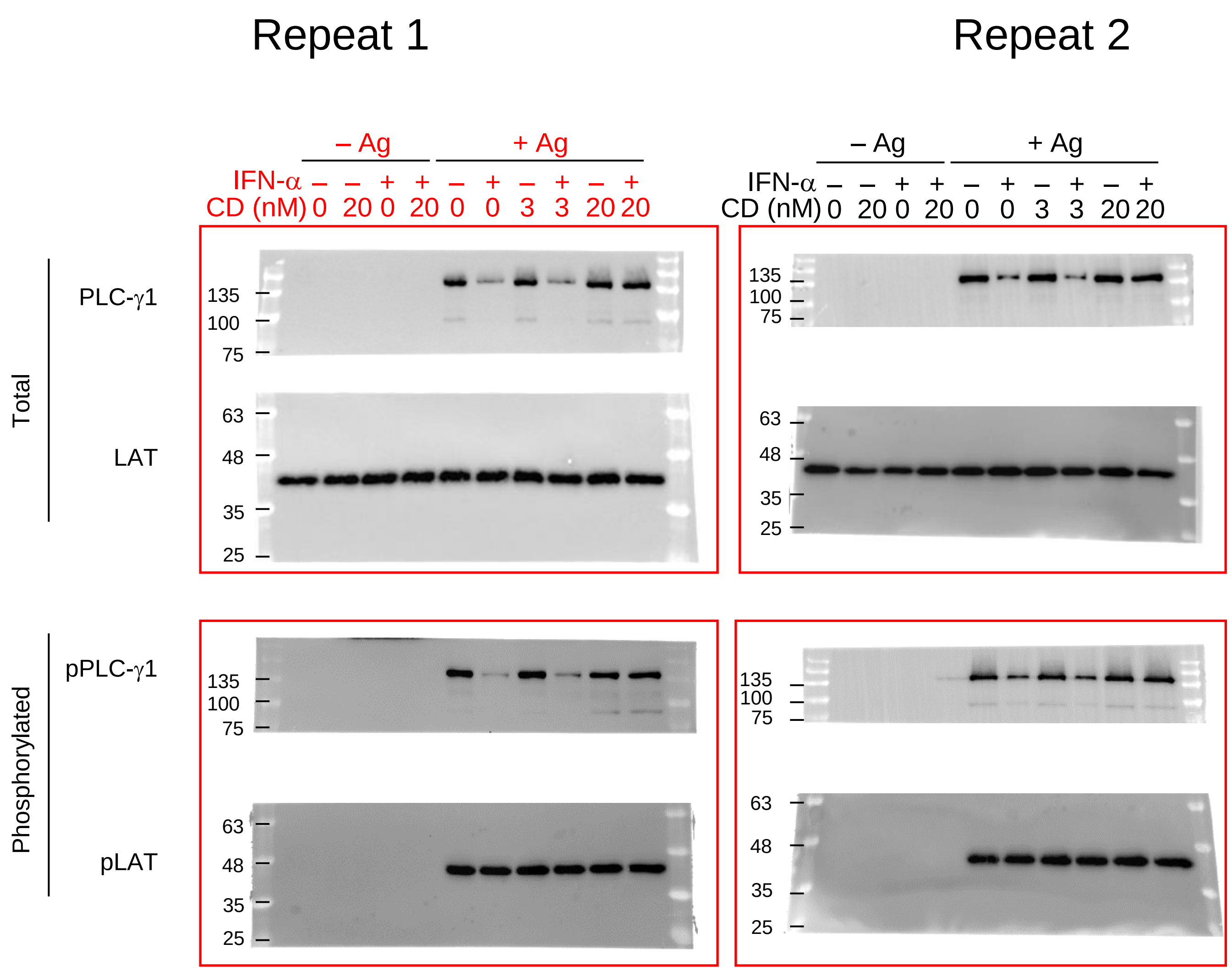

Supplement: Supplementary Materials [file mmc8.pdf]
